# Kinematics of the Cervical Spine Under Healthy and Degenerative Conditions: A Systematic Review

**DOI:** 10.1007/s10439-022-03088-8

**Published:** 2022-12-10

**Authors:** Sara Lindenmann, Christos Tsagkaris, Mazda Farshad, Jonas Widmer

**Affiliations:** 1 Spine Biomechanics, Department of Orthopaedics, Balgrist University Hospital, Zurich, Switzerland; 2 Department of Orthopaedics, Balgrist University Hospital, Forchstrasse 340, 8008 Zurich, Switzerland

**Keywords:** Motion, Flexion, Extension, Axial rotation, Lateral bending, Center of rotation, Range of motion

## Abstract

Knowledge of spinal kinematics is essential for the diagnosis and management of spinal diseases. Distinguishing between physiological and pathological motion patterns can help diagnose these diseases, plan surgical interventions and improve relevant tools and software. During the last decades, numerous studies based on diverse methodologies attempted to elucidate spinal mobility in different planes of motion. The authors aimed to summarize and compare the evidence about cervical spine kinematics under healthy and degenerative conditions. This includes an illustrated description of the spectrum of physiological cervical spine kinematics, followed by a comparable presentation of kinematics of the degenerative cervical spine. Data was obtained through a systematic MEDLINE search including studies on angular/translational segmental motion contribution, range of motion, coupling and center of rotation. As far as the degenerative conditions are concerned, kinematic data regarding disc degeneration and spondylolisthesis were available. Although the majority of the studies identified repeating motion patterns for most motion planes, discrepancies associated with limited sample sizes and different imaging techniques and/or spine configurations, were noted. Among healthy/asymptomatic individuals, flexion extension (FE) and lateral bending (LB) are mainly facilitated by the subaxial cervical spine. C4–C5 and C5–C6 were the major FE contributors in the reported studies, exceeding the motion contribution of sub-adjacent segments. Axial rotation (AR) greatly depends on C1–C2. FE range of motion (ROM) is distributed between the atlantoaxial and subaxial segments, while AR ROM stems mainly from the former and LB ROM from the latter. In coupled motion rotation is quantitatively predominant over translation. Motion migrates caudally from C1–C2 and the center of rotation (COR) translocates anteriorly and superiorly for each successive subaxial segment. In degenerative settings, concurrent or subsequent lesions render the association between diseases and mobility alterations challenging. The affected segments seem to maintain translational and angular motion in early and moderate degeneration. However, the progression of degeneration restrains mobility, which seems to be maintained or compensated by adjacent non-affected segments. While the kinematics of the healthy cervical spine have been addressed by multiple studies, the entire nosological and kinematic spectrum of cervical spine degeneration is partially addressed. Large—scale *in vivo* studies can complement the existing evidence, cover the gaps and pave the way to technological and clinical breakthroughs.

**Supplementary Information:**

The online version contains supplementary material available at 10.1007/s10439-022-03088-8.

## Introduction

One of the main functions of the cervical spine is to facilitate and control the motion of the head in a three-dimensional manner.^1,2,8,10^ Interrupted kinematics of the neck can cause clinical symptoms that we want to be able to diagnose and treat appropriately.^3,6^ The assessment of the kinematics of the cervical spine therefore is of great interest and reason for many studies in the last decades.^4,6,7^ Knowing the kinematic behavior can help clinicians identify numerous spinal diseases as well as promote improvement to motion sustaining products and prosthesis.^1,2,10^ The mobility can be characterized as general mobility of a whole spinal segment or between two consecutive vertebrae. The latter is especially important for new artificial disc replacement designs that aim to restore the natural movement of a motion segment and require precise knowledge of their relations. In the same manner we need to be able to differentiate between normal and abnormal motion to know when we are dealing with a spinal disorder.^1,2,4,5,10^ This knowledge can also help surgeons in preoperative planning, in conditions such as degenerative spondylolisthesis, which accounts for the largest proportion of these procedures and associated operative time and cost.^12^

Studying spine kinematics is not without challenges. In the literature, the described methods for studying physiological and pathological motion patterns span from simple static end-position image comparison to sophisticated techniques like 3D motion reconstructions.^70^ Performing these measurements in either laboratory or clinical settings adds up to the diversity of the results.^83^ Summarizing the relevant knowledge, identifying the knowledge gaps and resolving contradicting outcomes is necessary for the integration of spinal kinematics in translational research and clinical practice.

This review attempts to provide an extensive overview on published literature in the field of cervical spine kinematics under healthy and degenerative conditions *in vivo*. Our aim was to show data in a comparable manner and point out existing gaps in literature. Based on the early work by White and Panjabi on the basics of spinal kinematics published in 1978,^82^ and a previous review by Widmer *et al*.^83^ related to lumbar spine kinematics, we analyzed the literature on the following topics about the healthy cervical spine: angular segmental contribution, maximal range of motion (ROM), coupling, center or rotation (COR) and phase lag (PL). In addition to that, publications on disc degeneration (DD), facet joint osteoarthritis (FJOA), facet joint tropism (FJT), spondylolisthesis (SL), ligament degeneration (LD) and paraspinal muscle degeneration (PMD) of the cervical spine are summarized.

## Materials and Methods

MEDLINE was searched with predefined keywords for each topic individually, leading to 221 angular segmental motion contribution (Fig. 9), 221 maximal ROM (Fig. 10), 221 coupled motion (Fig. 11), 129 Center of Rotation (Fig. 12) and 193 Phase Lag (Fig. 13) records. Similarly, 162 DD—Mechanical Stiffness (Fig. 14), 2160 DD—ROM (Fig. 15), 22 DD—COR (Fig. 16) and 146—Spondylolisthesis (Fig. 17) publications were found for our section on pathologies. These results were then narrowed with inclusion and exclusion criteria and supplemented with a reference search. All papers from 1980 to 2021 were included. Cadaveric studies were excluded, since they neglect neuromuscular interaction, which is one of the most essential components defining spinal in-vivo behavior.

**Figure 1:**
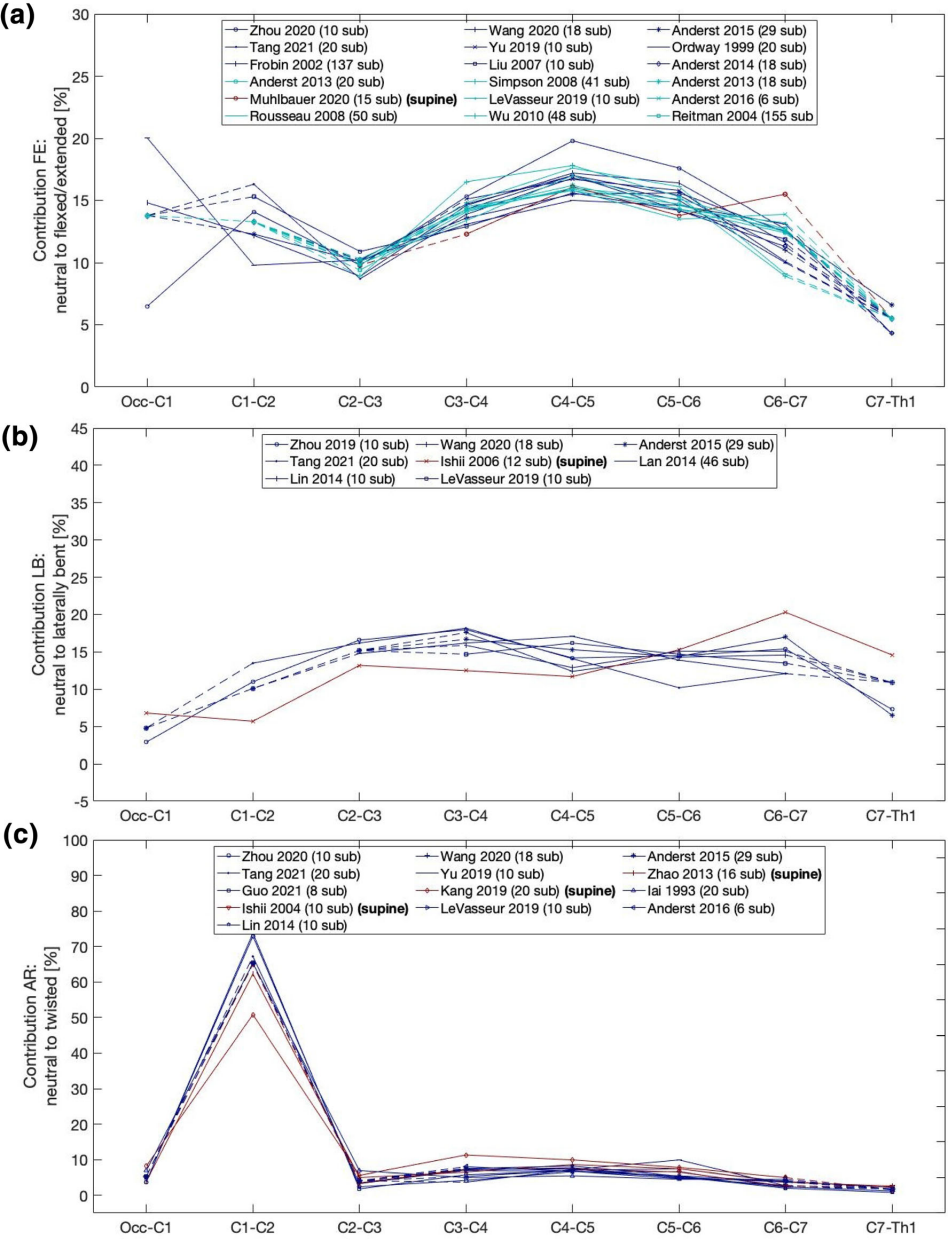
Angular contribution of the segments Occ-Th1. (a) Flexion/Extension; (b) Lateral Bending; (c) Axial Rotation. Dashed lines are used when the values for certain levels were not available and an average value from the other studies was used. Studies conducted in a supine position of the subjects are marked in red.

**Figure 2:**
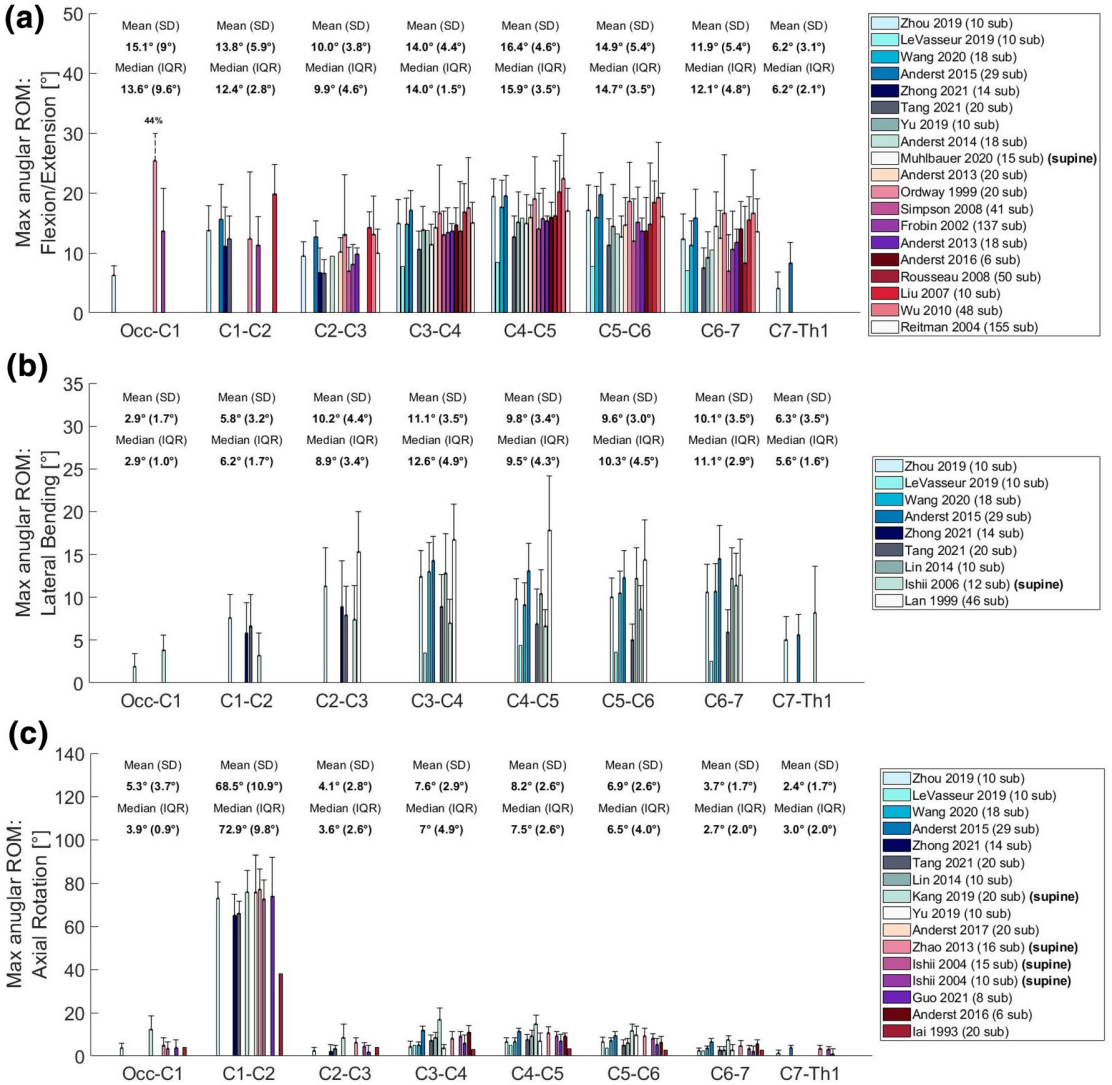
Maximal angular ROM for each cervical segment between Occ-Th1 expressed in [°]. (a) Flexion/Extension; (b) Lateral Bending; (c) Axial Rotation. For every segment the mean, SD, median and IQR were calculated based on the available values. Error bars represent the standard deviation (error data was not available for data from Anderst *et al*. 2015^6^, LeVasseur *et al*.^48^ and Iai *et al*.^35^).

**Figure 3:**
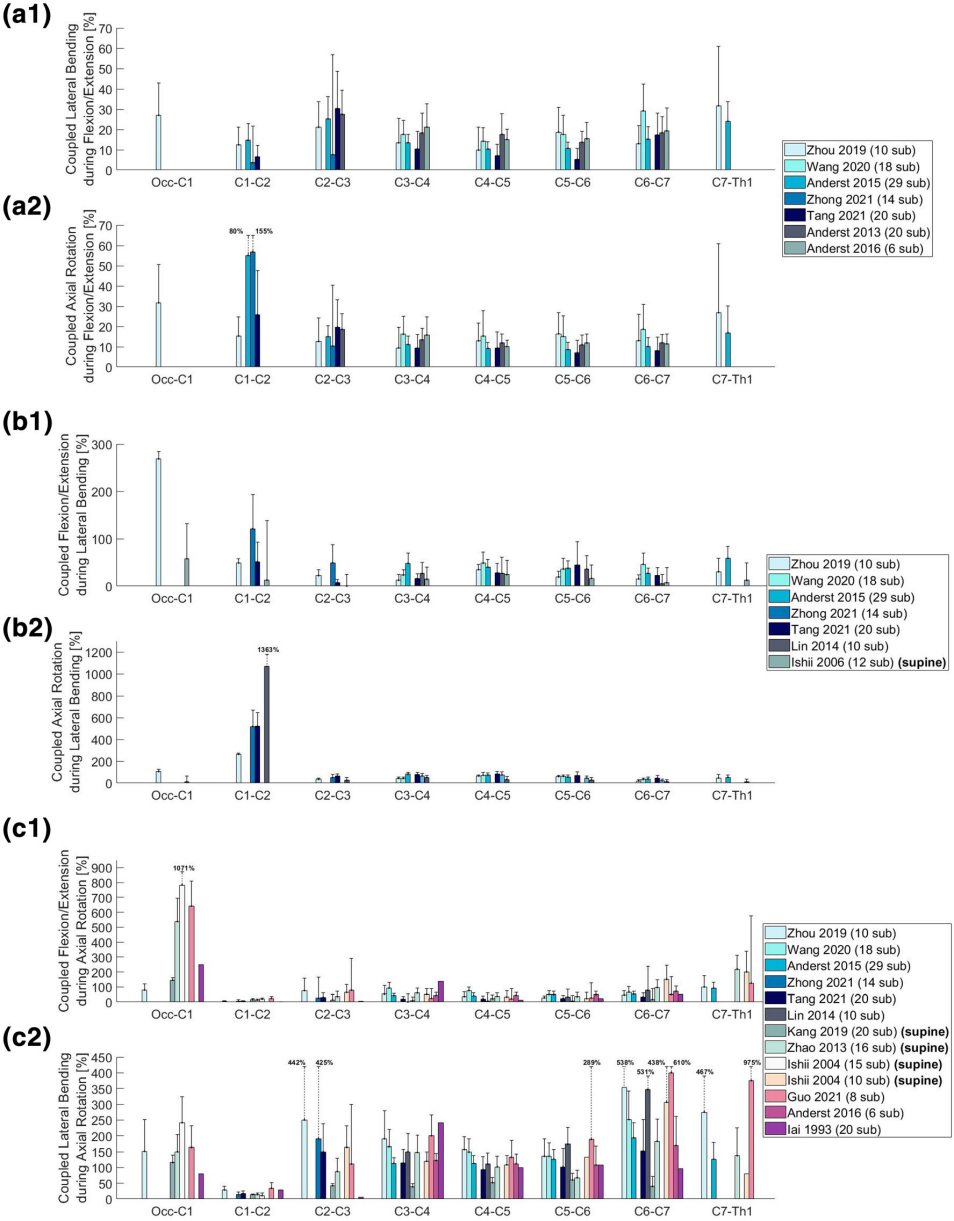
Coupled rotational motion (as ratio [%] of the maximal ROM of the primary motion). (a) Coupled rotational motion during flexion/extension; (b) Coupled rotational motion during lateral bending; (c) Coupled rotational motion during axial rotation. Values exceeding 100% are larger than the movement in the primary direction.

**Figure 4:**
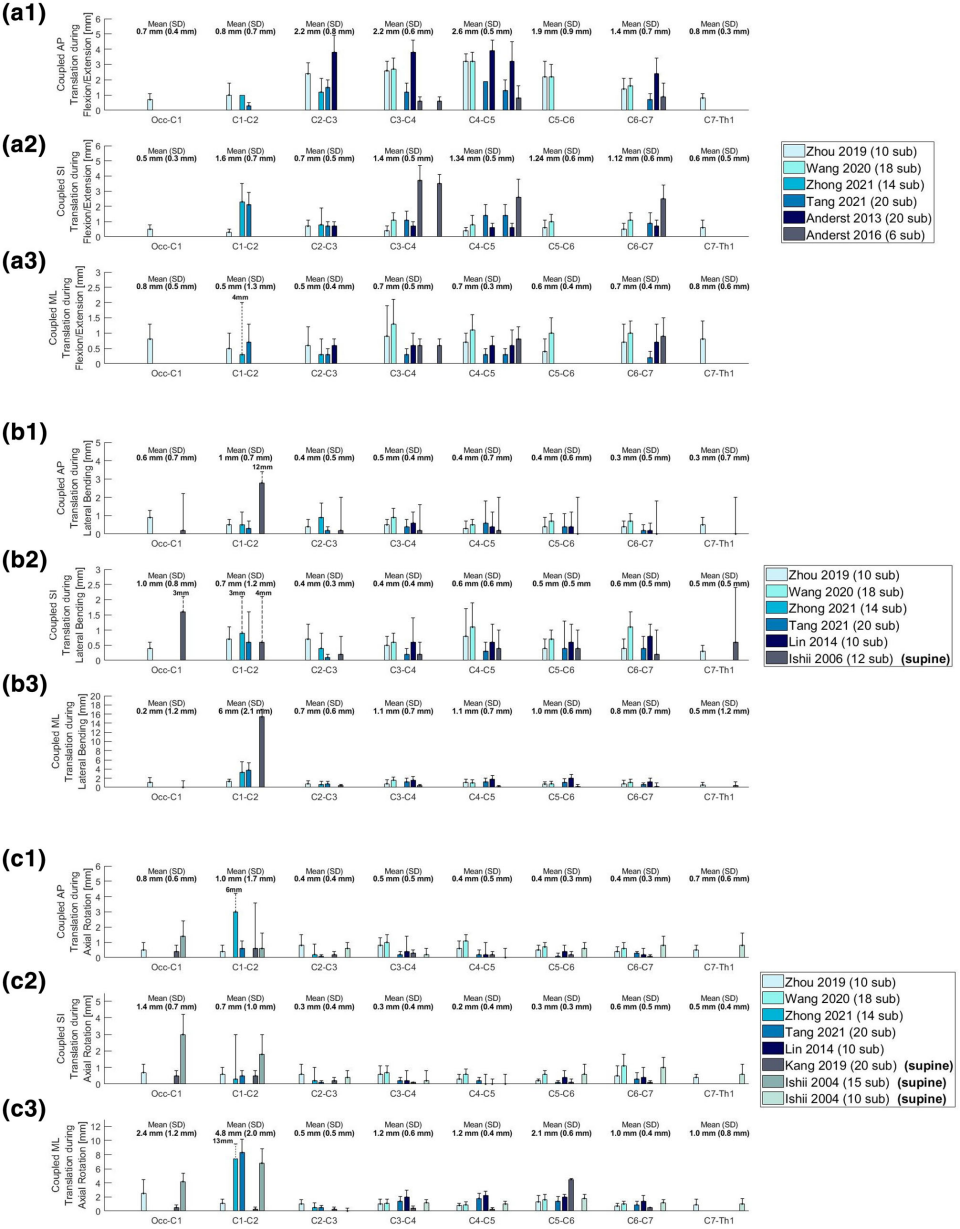
Coupled translational motion in anterior–posterior (AP), superior-inferior (SI) and medial–lateral (ML) direction (expressed in mm). (a) Coupled translational motion during flexion/extension; (b) Coupled translational motion during lateral bending; (c) Coupled translational motion during axial rotation.

**Figure 5:**
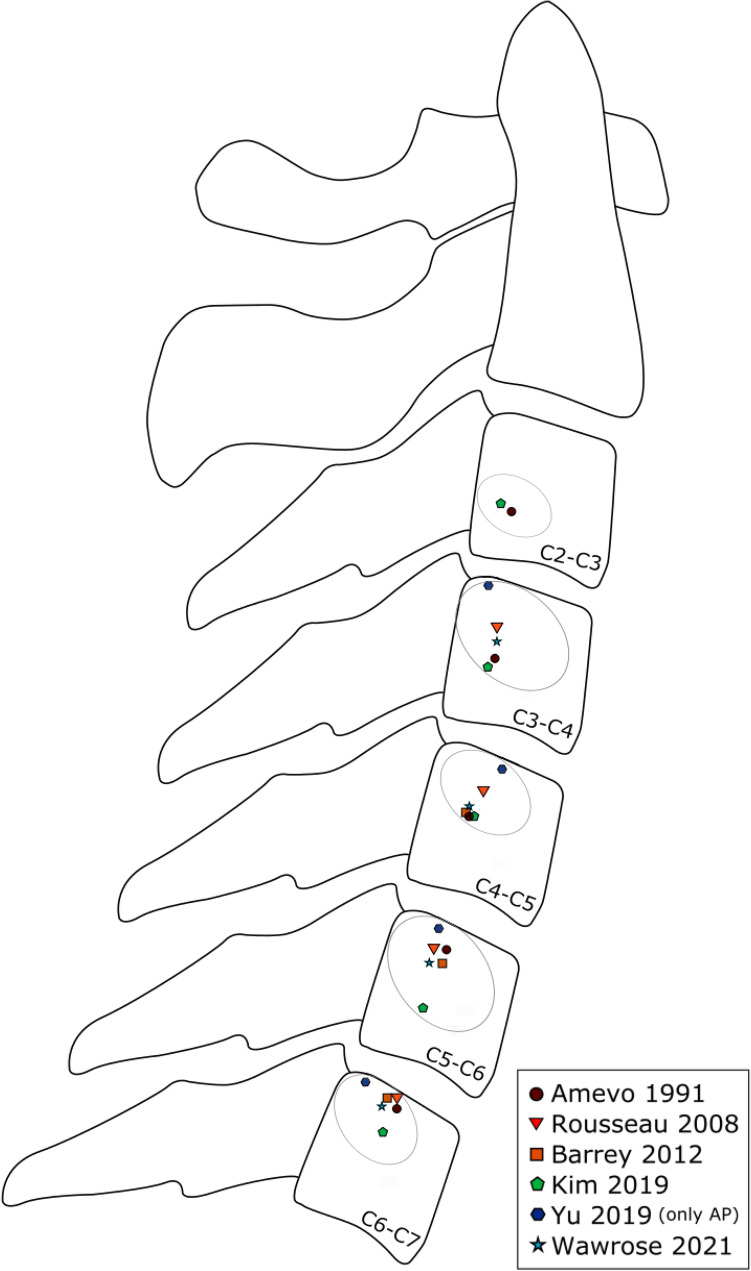
Center of rotation as observed in studies involving healthy participants and providing data eligible for inclusion in a figure.

**Figure 6:**
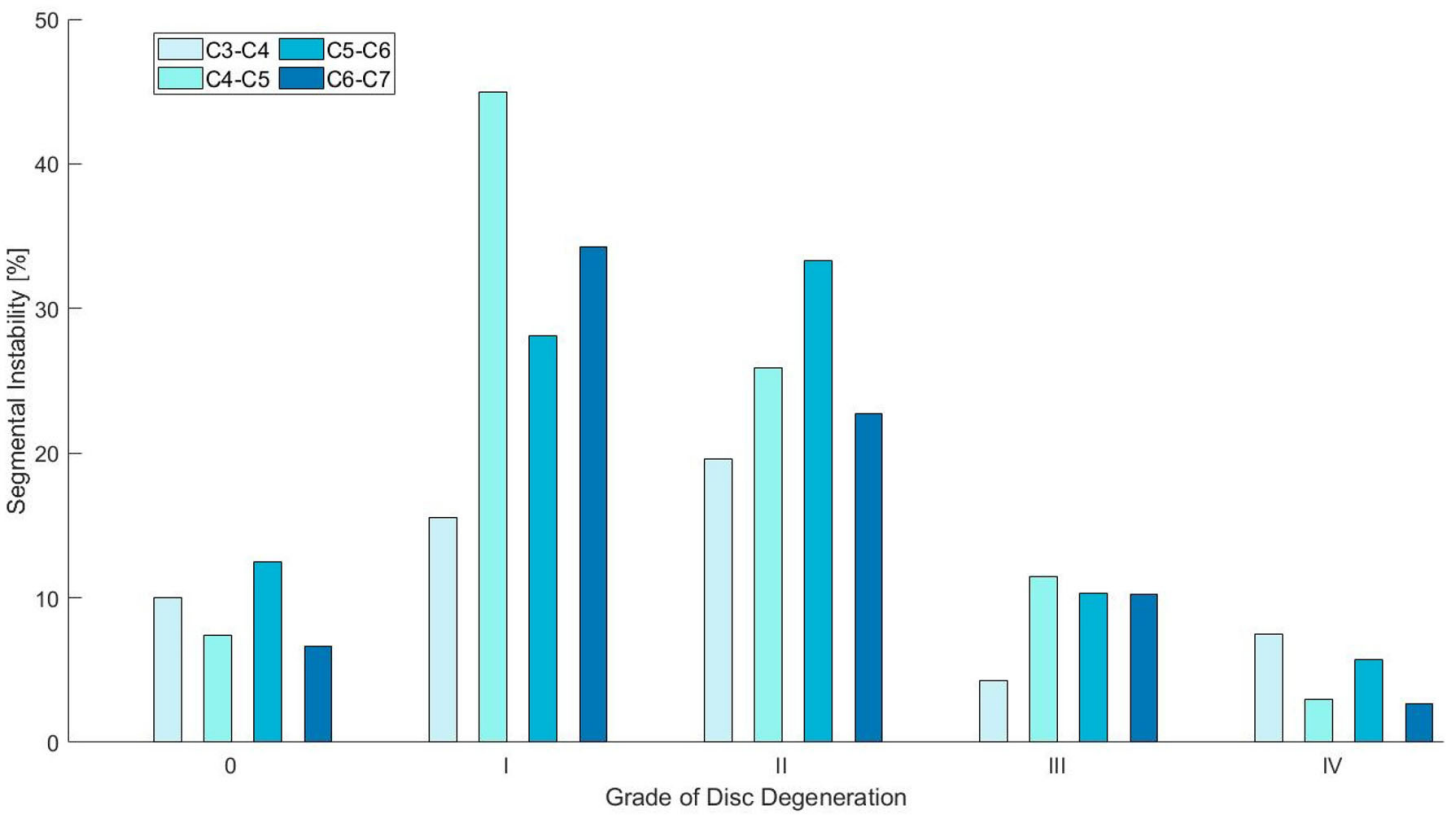
Portion [%] of segmental instability based on the grade of disc degeneration in the intervertebral levels C3–C7. Cervical segmental instability was considered when the sagittal plane displacement between two cervical vertebrae was more than 3.5 mm or the relative sagittal plane angulation was greater than 11°. The illustrated data is from Dai *et al*.^21^ and comply with the five-grade DD scale of Mehalic *et al*.^54^

**Figure 7:**
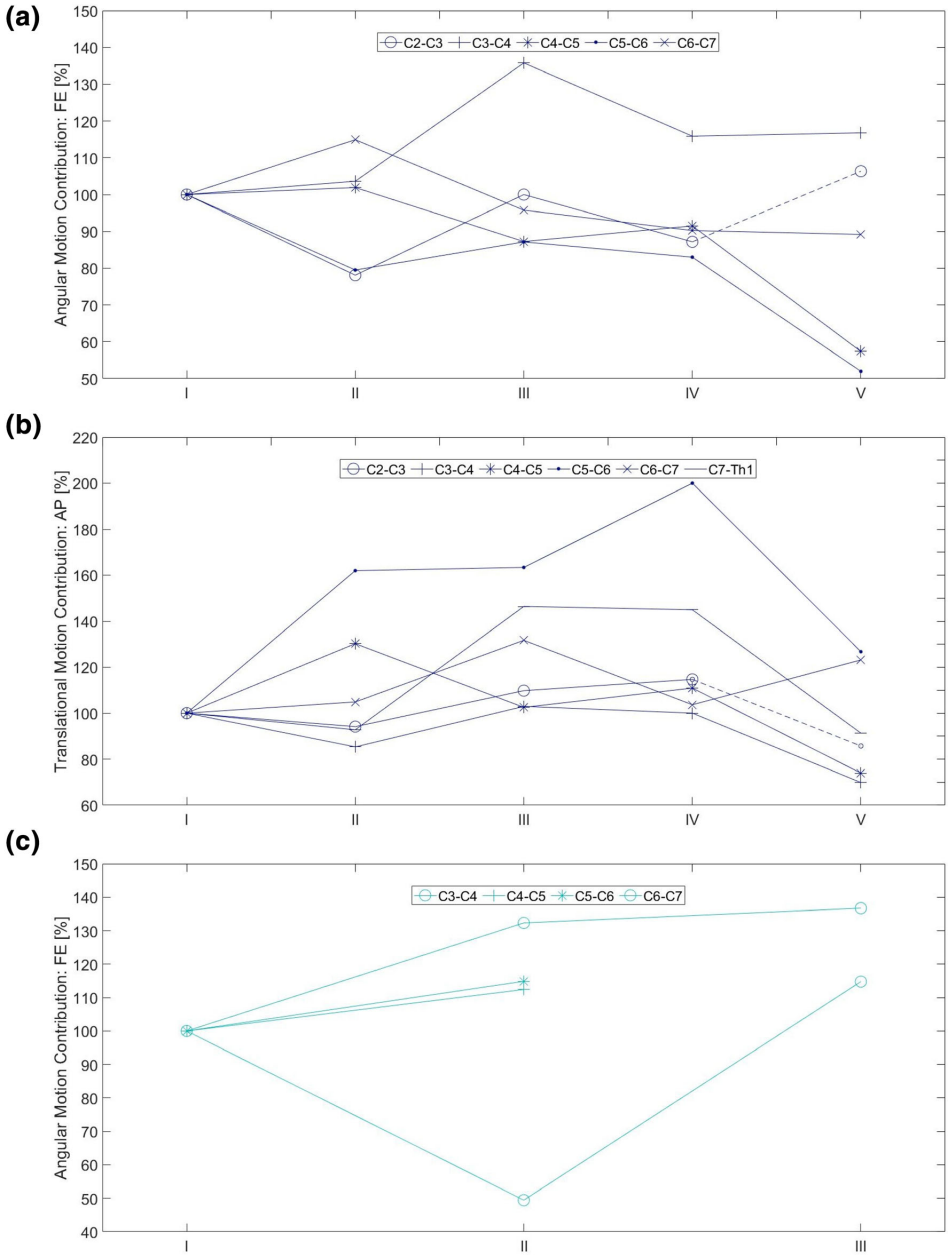
Angular motion (a) and translational motion (b) contribution for Flexion–Extension in relation to the degree of disc degeneration according to the data by Miyazaki *et al*.^56^ They used their own DD grading system with five levels of disc degeneration. Motion contribution is expressed in % to the total motion from C2 to C7 and C2 to Th1 respectively. (c) Angular motion contribution (in % to the total motion from C3–C7) for Flexion–Extension in relation to the degree of disc degeneration according to the data by Morishita *et al*.^57^ They used their own DD grading system with three levels of disc degeneration. I, II and III correspond to the level of cervical cord compression defined by Morishita *et al*.^57^ All the measurements are normalized to 100% to make comparison easier. For instance, angular motion contribution of 130% for C4–C5, implies that the segment contributes 30% more than expected in its healthy configuration.

Eventually, this resulted to a final selection of 25 Angular segmental motion contribution (Fig. 1; Table 1), 30 maximal ROM (Fig. 2; Table 2), 15 Coupling (Fig. 3; Table 3), 9 COR (Fig. 4; Table 4) and 1 Phase Lag (Table 5) publications. 1 DD—Mechanical Stiffness (Fig. 5; Table 6), 4 DD—ROM (Fig. 6; Table 7), 2 DD—COR (Table 8) and 1 Spondylolisthesis (Fig. 7; Table 9) studies were selected for the part on pathologies. A detailed description of the applied search strategy and precise literature overview is provided in the Appendix.

Results of the final literature selection were brought into a comparable form. All results dealing with segmental contribution were converted into percentages, relative to the overall motion from C1-Th1. When data was missing for certain segmental levels, an average value from all available percental values on that level was taken and visualized with a dashed line. Results that were separated into groups according to age, sex or other factors were averaged. For left and right LB and AR pooled values were used. Additionally, statistical error propagation was considered when pooling standard deviation values.^25^

## Healthy Conditions

The cervical spine bears the weight of the head and is vital for its physiological function. Vision, hearing, smell and taste greatly dependent on the movement of the neck.^17^ The kinematic analysis of active cervical spine motion involves a number of parameters, namely the segmental motion contribution, the maximal range of motion (ROM), coupled motion, the center of rotation (COR) and phase lag.

The reported studies have used various two- (2D) and three-dimensional (3D) methods in order to collect relevant information for healthy volunteers and in some cases patients. The most popular techniques include lateral or dual neck radiographs (*n* = 13), fluoroscopy (*n* = 9), computed tomography (CT) (*n* = 15), magnetic resonance imaging (MRI) (*n* = 6) or combinations of the above (*n* = 12). Due to the nature of the examinations, measurements were obtained in standing, supine or sitting configuration. The latter renders the comparison between measurements obtained in different postures weaker due to the alterations in spinal alignment and in the muscular and connective tissue.^46^

All the included studies focused on active movement, studies reporting passive movement were excluded because they do not provide evidence regarding functional movement patterns, which affect healthy individuals in their everyday lives.

### Segmental Motion Contribution

The cervical spine is the most mobile part of the vertebral column. Its overall mobility is the sum of smaller movements of all the segments from the atlas to Th1. Segmental motion contribution represents the percentile contribution of its segment to overall cervical mobility and varies considerably across levels of the cervical spine and performed movements (flexion extension, lateral bending, axial rotation).^11,41,93^

The majority of the included studies were based on CT or MRI oftentimes combined with (video)fluoroscopy^29,50,80,88,93^ or biplanar radiographs.^3,6,7,9,11,88^ Fewer studies were based solely on radiography^27,35,71,75^ or fluoroscopy.^52,70,85^

### Flexion/Extension

FE was reported in 18 studies, the majority of whom contained measurements from Occ-C1 to C7-Th1 (except from Anderst, 2014^6^ and 2013^3,9^) (Fig. 1a). The cervical FE range resembles a parabolic motion as a result of the composition of a uniform rectilinear motion (represented by the greatly immobile thoracic spine) and a uniformly accelerated rectilinear motion of launching upward or downward (represented by the head). Segmental contribution to FE spanned between 11 and 20% for all segments with the lowest contribution at C7-Th1 that contributed between 3.3 and 6.6% of the motion. This is attributed to the articulation between C7 and the hardly mobile thoracic spine. A cranio-caudally decreasing contribution trend was observed from Occ-C1 to C2–C3 and from C3/C4 to C6/C7, with Occ-C1 and C3–C4 contributing up to 20% of the cervical FE. In absolute numbers, the reported segmental motion contributions spanned from 2.1 to 13.8°. C4–C5 and C5-6 were the major FE contributors in all the reported studies. However, the FE contribution of C6–C7 was greater than the contribution of C4–C5 and C5–C6 in the supine position.^58^ This explains why C3–C6 fusion results in major FE reduction.^86^ Most importantly, this differentiation implies the need for an additional point of FE mechanical support to compensate the supine allocation of the head's gravitational force. (see Fig. 1a).

Although most studies reported initial and end positions,^27,58,61,71,75,78^ some evidence regarding the dynamic implications of FE occurring between the documented endpoints was recorded. It appears that between C2–C3 and C7-T1, the helical axis of motion nearly adhered to the sagittal plane,^7^ with the extent of angular motion observed in FE being larger than angular motion in left – right movement of the neck. Angular motion accompanying FE was rather segment – dependent.^7^ Hence, intersegmental rotation during FE was limited and the maximal ROM of adjacent segments did not increase over time.^11,52^ These findings can be coexamined with the anatomy of the cervical spine. The odontoid dents articulation in combination with the restrictive effect of the tectorial membrane can explain the gradual decrease in FE segmental motion contribution from Occ-C1 to C2/3. In lower cervical segments, the increasing inclination angle of the facet joints can limit FE.^39^ Simultaneously, the partial thinning or even lack of articular surface between the upper cervical facet joints can result in these segments flexing or extending few more degrees than the lower segments.^87^

### Lateral Bending

8 studies reported on LB. The highest level of contribution, ranging between 11.7 and 20.3% was observed between the C3–C4 and C6–C7 segments (Fig. 1b). The contribution of these segments to LB was homogeneous ranging from 12 to 17% at each level. The cranial and caudal extremities of the cervical spine had minor contribution to LB, presumably due to their strong attachment to the head and the thoracic spine respectively. Ishii and colleagues contradicted the aforementioned reporting significantly higher LB contribution between C6 and Th1 (20.3 and 14.6% respectively). The measurements used in the study were acquired in supine position, making the comparison between this and the remaining studies weak. Nevertheless, the supine position has been associated with a lower level of activity of the right scalenus and the trapezius muscle, which can explain the decrease in LB at the C3–C4 and C4–C5 segments.^46^ On top of this, the increased contribution of the C6–C7 segment to LB in this position may indicate the anterior and superior shift of the center of rotation observed at this level.^1,81^ (see Fig. 1b).

### Axial Rotation

13 studies included data about segmental motion contribution to AR (Fig. 1c). In contrast to FE and LB, AR is predominantly achieved at the C1–C2 segment, which contributes between 63 and 73% of this type of movement. Axial torque leads to greater rotation at this segment because of the atlantoaxial articulation, which does not restrict movement at this plane. Segments between C3–C4 and C5–C6 appear to account for 5 to 11% of neck's AR, while the contribution of the remaining segments was limited to 5% or less in most cases. In absolute numbers, the C1–C2 AR spanned from 38.5° to 65.8°, while the least contributing segments would rotate only by 0.4 to 7.3°. This is consistent with studies on 3D cervical spine models reporting > 25% length alterations for local spine ligaments, namely the occipito-atlantal, atlanto-axial and apical ligaments.^16^ Studies with measurements obtained in supine position^37,38,41,91^ did not yield different trends than the rest of the studies with regard to segments between C2–C3 and C7-Th1. A different pattern was observed at the C1–C2 level, which had an up to 30% lower contribution to AR in supine position. This was moderately compensated at the C3–C4 and C4–C5 segments, which contributed 5–10% more AR than non-supine configurations.^37,38,41,91^ Both the decreased dynamic loading from the segments' musculature and the different allocation of the head's weight can be associated with this.

### Maximal Range of Motion

ROM was analyzed with regard to total flexion and extension (FE), lateral bending (LB) and axial rotation (AR). The majority of studies focused on FE (*n* = 19), mainly between C3 and C7 (*n* = 18) (Anderst, 2013^3,5^ is the exception), and in some cases between the occipital bone and Th1,^93^ or C1–C2 and C7-Th1^7^ and C1–C2 and C2–C3^92^ (Fig. 2a). Most accounts regarding LB focus on C3–C7 (*n* = 9, apart from^92^) and fewer include data about Occ-C3 and C7-Th1^37,38,78,93^ (Fig. 2b). AR was commonly reported between C3–C7 (*n* = 13) while fewer studies referred to Occ-C1, C6-Th1^29,41,91,93^ or C1–C3 (Fig. 2c).

The mean FE ranged between 10.0 and 16.4° in all IVS apart from C7-Th1, where FE motion was restricted to 6.2°, due to the connection with the relatively immobile thoracic segment. The mean LB was high, ranging between 9.8° and 11.1°, from C2–C3 to C6–C7, low in C1–C2 and C7-Th1 (5.8° and 6.3° respectively) and lowest in Occ – C1 (2.9°) For the same reasons, the mean AR was higher among C2–C3 and C6-7, where it ranged between 10.2° and 11.1°. It is significantly lower in C1–C2 (5.8°) and Occ-C1 (2.9°), because of the rigid connection between the occipital condyles and the atlantal sockets.

The majority of the studies were consistent for most observations with SDs ranging between 1.7 and 4.6° and IQRs ranging between 2.1 and 4.9°. Slightly higher deviations (SDs 5.4°–5.6°) were observed in the reported values for FE in C1–C2, C5–C6 and C6–C7. Large deviations were observed in FE in Occ-C1 (SD: 9°, IQR: 9.6°) and AR in C1–C2 (SD: 10.9°, IQR: 9.8°). In the first case, there were only three available studies.^27,61,93^ The discrepancies can be attributed to imaging methods, the objectives and the studies' populations. The older studies reporting higher Occ-C1 FE, were based on hand or computer measurements on lateral neck radiographs,^27,61^ while Zhou *et al*. (2020) used dual fluoroscopic imaging.^93^

Biomechanical factors that may affect the variations in cervical ROM include, facet joints and IVDs' height. Facet joints' angular configuration is estimated at 45° on average, increasing from 20 to up to 78° between C1 and C7. At the same time, the facets' plane changes from posteromedial to posterolateral at the C5–C6 level, which sustain the highest rate of disc degeneration in the cervical spine.^24^ FE and AR reach their maximal ROM at the C4–C5 level indicating that a relatively increased angulation of facet joints in posteromedial plane is the most favorable towards wide ROM. Nevertheless, the posterolateral facets' plane facilitates the highest LB ROM at the C6–C7 level.^63^ It is also possible that the integrity and thickness of adjacent soft tissue, namely the capsule surrounding the facet joints, can affect ROM, however relevant in silico studies have failed to establish a significant correlation.^20,31^

The height of non-degenerated IVDs ranges between 5.57 mm and 4.94 mm in radiography and computed tomography respectively according to a study conducted in Korean individuals.^19^ The mean disc height at C2–C3 and C6–C7 was lower compared to the segments between them. The same pattern applies to the FE ROM between C2 and C7 (Fig. 2a); the reported maximal FE ROM for C2–C3 and C6–C7 are lower than those of the segments between them. Hence, it is possible that greater disc height allows more angular motion in FE.

Overall, maximal ROM has been observed between C4 and C6. Studies on healthy individuals were greatly consistent and a limited number of discrepancies noted can be attributed to differences in image acquisition and measurement. The current biomechanical interpretation of cervical ROM is based on facet joints and IVDs, nevertheless the contributions of other structures, namely ligaments and muscles should be further investigated.

### Coupled Motion

From a structural point of view, the curvy configuration of the spine in combination with its static and dynamic support from ligaments and muscles renders pure uniaxial motion impossible. Therefore, cervical spine motion around one axis is coupled with translational and rotational movement around or across other axes and indeed the primary motion cannot happen unless it is accompanied by the secondary motion. Flexion – Extension (FE), lateral bending (LB) or axial rotation (AR) can act as primary movement. During rotation, spinning or angular displacement of vertebral bodies around a different axis than the one who initiated the motion can occur. During translation, FE, LB or AR primary motion is coupled with segmental motion of the same direction and velocity towards the anteroposterior (AP), mediolateral (ML) or superior – inferior (SI) axis.^41,83^

All the included studies assessed cervical active coupled motion in healthy subjects by means of CT or MRI alone or combined with fluoroscopy. Iai *et al*. (1993)^35^ based their assessment on radiographies, which were subsequently deemed inappropriate for the accurate representation of vertebral orientation during dynamic motion.^9^ The majority of the studies examined individuals in upright standing positions, while a number of them focused on supine^36–38,41^ or seated position.^50^ All the studies examined the subaxial C2–C3 to C6–C7 segments, with fewer studies providing evidence about Occ-C1,^93^ C1–C2^7,78,92,93^ and C7-Th1.^7,93^

#### Angular

As a primary motion, FE exerted a light to moderate increase in LB and AR from C2–C3 to C6–C7 (Fig. 3; a1, a2). LB was significantly increased at C2–C3 in all studies (apart from^7^) and reached to up to 30% of the coupled motion at the Occ-C1 and the C7-Th1 level. Notable increases in coupled AR were observed at the C1–C2 level, where it almost reached^7^ or even surpassed^92^ the contribution of FE to the total motion achieved. All the studies examined active movement of the neck in standing position and subject (age, posture) or observer—associated factors can be considered as the main source of the variations observed.

During LB, the coupled contribution of either FE or AR was relatively small and homogeneous in the subaxial cervical spine (Fig. 3b1 and 3b2). Excessive coupled motion was observed at the Occ-C1 level (ca 270%^93^) for FE and at the C1–C2 level (ca 1360%^50^) for AR. The authors of the former attributed the difference to C0-1 articulation anatomy of the superior cervical through cartilaginous interface joints without intervertebral discs, which favor FE but constrain LB and AR.^93^ The variation in the latter can be attributed to the different distribution of the gravitation force of the head to the seated spine^50^ and the almost straight cervical configuration in the same position.^73^

During AR, excessive coupled FE is observed at the Occ-C1 and C7-Th1 level (Fig. 3c1 and 3c2). While the rigid Occ-C1 articulation accounts for the predominance of FE at the superior cervical spine, at the C7-Th1 level, similar effect is yielded by the adjacent immobile thoracic segments.^93^ On the contrary, a pattern of coupled LB exceeding by 2–6 times the range of AR is common in the entire cervical spine, with a notable exception at the C1–C2 level, presumably associated with the local stabilizing effect of the alar ligament.^41^ The largest percentages of coupled LB were observed at the C6–C7 level, which is significantly more mobile than its caudal adjacent segment whose mobility is limited by the thoracic vertebrae. The largest percentages of coupled LB were mostly observed in studies involving individuals^37,38^ in supine position, where the gravitational vectors of head’s weight are shifted towards the MRIs table and the spine needs to develop compensatory mechanisms, so as to support the head.

#### Translational

In the course of FE, significant coupled AP, SI and ML translation (exceeding an average of 0.5 mm) is observed between C2–C3 and C6–C7 (Fig. 4; a1, a2, a3). A similar pattern of SI translation is observed at the C1–C2 level. The highest AP and SI, translations were observed in the studies of Anderst *et al*. in 2013^9^ and 2016^11^ respectively. During LB, minimal translation patterns – rarely exceeding 1.5 mm – have been observed in the subaxial segments. On the contrary, a marked increase in AP and ML translation was observed at the C1–C2 level (12 and 15 mm respectively). Relatively high SI translation was also observed at the Occ – C1 level (Fig. 4; b1, b2, b3). During AR, translational patterns inferior to C1–C2 rarely exceeded 2 mm at any axis – with an exception for the study of Kang, 2019, where 5 mm ML translation was observed at the C5–C6 segments of individuals placed in supine position. In this position, it was observed that Occ to C5 segments were all coupled with the back-extension movement (Fig. 4c1, 4c2, 4c3).^41^

Nevertheless, significant AP and ML translation was observed at the C1–C2 level and similarly notable SI translation was observed at the Occ-C1 (Fig. 4; c1, c2, c3). More translation occurs at the Occ-C1 and C1–C2 level than in the subaxial spine. It appears that the limited capacity of these segments to rotate due to their rigid interconnection, favors translation as a response to mechanical loading. Within the superaxial spine, ML and AP translation occur mostly at C1–C2 level, while SI translation occurs mostly at the Occ-C1 level. It appears that the attachment of the alar ligaments to the lateral and posterolateral aspects of the odontoid process limits the C1–C2 SI translation. The C1–C2 AP and ML translation may be a combined result to its cranial rigid attachment of the axis to the atlas and its less rigid attachment to the caudal adjacent segment. The latter enables more translation than the one observed at the Occ-C1 level.

From a quantitative point of view, translation occurs less than rotation, particularly in the subaxial cervical spine, where the translation – rotation index has been found to range between 0.013 and 0.017. This indicates that the main purpose of translations was to maintain the balance between stability and mobility of the cervical spine.^41^

### Center of Rotation

The observations regarding center of rotation (COR) are presented in Fig. 5. The perception of the COR has evolved during the last two decades in parallel to the methods and findings of relevant research. The whereabouts of the concept can be traced to clinical observations deeming the determination of the instantaneous axes of rotation (IAR) necessary for the clinical evaluation of subtle abnormalities in cervical motion.^1^ Its determination during flexion—extension was agreed upon as “a pair of coordinates offset from the posterior superior corner of the lower vertebral body”.^15,58,71^ The latest studies on the matter focus on the instant center of rotation (ICR), which is defined as “the point at which the three-dimensional axis of rotation vector intersected the sagittal anatomical plane of the inferior vertebra”.^2,81^ Some studies narrow this investigation down to the anteroposterior (AP) direction,^88^ while others provide a circular estimate of possible distribution areas of ICR in the sagittal plane at each level in the course of *in vivo* movements.^42^

Studies published before 2012^1,15,22,71^ were based on radiographs taken between the initial and final position of the studied movements, while studies published later employed a combination of computed tomography (CT) or magnetic resonance imaging (MRI) with biplanar radiography or (video)fluoroscopy.^2,42,58,81,88^ The former predominantly report a stationary ICR location at each cervical segment, which derives from the average of measurements collected from static full-flexion and extension radiographs and from radiographs collected during slow sagittal movement.^14^ The techniques used by the latter group of studies provide a more accurate account about motion of the ICR during the dynamic flexion–extension cycle and identify bone location and three-dimensional orientation with sub-millimeter accuracy.

*In vivo* studies involving human subjects confirm that from C2–C3 to C6-7, the centers of rotation move superior for each successive segment.^1,2,22^ For C2–C3, the center of rotation is located in the middle of body, and for C6–C7, it is located on the superior end-plate .^22^ This was observed at a higher level in the study of Amevo *et al*. (1991), where the center of rotation shifted towards the middle of the vertebral body from C4–C5 onwards and could even be observed at the inferior part of the C2–C3 vertebral body.^1^ The COR gradually moved anteriorly from C2–C3 to C6–C7^1,22^ and superiorly in all subaxial segments apart from C5–C6.^42^ The antero-posterior (AP) change decreased per degree of flexion–extension,^2,42,88^ although it appears that the position of the CORs is more posteriorly inclined in left–right-left (LRL) rotation than in flexion – extension.^88^

A number of deviations were observed in the reported findings. Although the spatial distribution of the reported CORs was highly homogeneous in the C3–C4 and C4–C5 segments, with an exception for the findings of Amevo (1991)^1^ and Rousseau (2008).^71^ Amevo positioned the COR, defined as IAR in the study, close to the C4 endplate, a deviation that may be explained due to the different imaging sampling methods and a number of poor radiographic images of the particular level that were excluded from the analysis of the study.^1^ Rousseau *et al*. (2008) positioned the COR anteriorly and inferiorly in all levels, a change associated with the fact that the half of the participants of the study has undergone ball-and-socket total disc arthroplasty.^71^ In the patients of the study, the flexion – extension COR was altered in the whole cervical spine regardless of the instrumented level(s). A similar trend was detected by Muhlbauer *et al*. (2020) who associated prostheses with ball-socket, inverse ball-socket design and prostheses with two surfaces with transition of the COR to any direction and at any level.^58^ On the contrary, the studies of Barrey *et al*. and Anderst *et al*. reported that arthrodesis had no significant impact on the location of the ICR or the change in ICR location per flexion – extension degree of any other level apart from the instrumented one.^2,15^ At the instrumented level, COR location was shifted cranially.^15^

Overall, it appears that in the subaxial cervical spine COR location moves inferiorly and anteriorly in the sequence of motion (cranially to caudally) with the AP change in COR decreasing per degree of flexion – extension. COR, and particularly its approach as ICR, is regarded as a stable, and thus credible, measure of the quality of vertebral motion,^14^ which can be recorded in healthy individuals and individuals undergone surgery. The latter seems to create a “new normal” in terms of cervical spine COR. Arthrodesis affects the quality of movement at a single level – such a discrepancy can contribute to or precipitate adjacent segment pathology by means of disc and endplate inflammation and degeneration. Solid prostheses with non-flexible biomechanical properties can entirely alter the cervical COR location. This may increase the needs for compensatory static loading from the cervical ligaments or dynamic loading from the cervical muscles and in time this may result in ligament and muscle injury and related clinical manifestations. In these cases, monitoring COR alterations can provide valuable insights regarding the pathomechanics of the concerned cervical conditions.

### Phase Lag

Phase lag represents the time interval observed between the transmission of motion across cervical segments. To the authors’ knowledge, this has only been investigated by one *in vivo* study based on cineradiography.^34^ Both longitudinal displacement and cervical angular motion were initiated at the C1–C2 level, and then transmitted stepwise towards C5-6. C2–C3 motion occurred 1.8 s after the start of cervical flexion, while C3–C4, C4–C5, and C5–C6 angular motion onsets occurred 2, 2.2 and 2.6 s after motion's initiation, respectively. Similar trends were observed in longitudinal displacement with a stepwise motion transmitted from C1-2 to C5-6 with a time lag. C1–C2 plays a key role in this type of motion. It appears that the rigid atlantoaxial junction necessitates more time for the initiation of movement (1 s) in comparison to the lower cervical segments, where the same time suffices for the transmission of motion from C2 to C4 and even less time (0.6 s) is required for the same between C4 and C6. The accelerated motion pattern between C3 and C6, can be associated with the structural and kinematic differences of the superior and inferior cervical group. In the former, the cradle structure of the atlantooccipital joint together with the stabilizing role of the alar ligament and the lack of muscle involvement in rotation are likely to pose biomechanical limitations to the initiation of movement. Even within the inferior cervical group, phase lag at the C2-3 level is significantly greater than phase lag at the lower levels (0.8 s as opposed to 0.2 and 0.4 s). This can be attributed to the limiting effect of the posteromedial orientation of the C2–C3 facet joint on segmental rotation.

From a clinical point of view, time lag reflects the spatiotemporal dimension of cervical mobility—the stepwise – sequential transmission of angular and longitudinal motion evolves across time. In this sense, and on the grounds of further research, deviations from the physiological time lag pattern may indicate an established pathology (injury, degeneration etc.) posing additional limitations to motion transmission or precipitate further pathomechanical alterations.

## Degenerative Conditions

Cervical spine degeneration is the most common cause of neck pain which burdens up to 35% of the global population. Although cervical spine degeneration rates increase with patient age, symptoms are common among individuals between 40 and 60 years and may manifest in people as young as 30 years.^72^ The diagnosis and classification of cervical spine degeneration remains challenging, since not only mechanical, but also systemic, environmental and genetic factors are implicated in the manifestation and progression of the disease.^24,53^ Degenerative conditions of the cervical spine are currently classified based on the degree of degenerative lesions in lateral spine radiographs^60^ or magnetic resonance imaging.^77^ Associating these conditions with particular pathomechanical motion patterns can improve their diagnosis and management.

### Disc Degeneration

The existing discourse on cervical disc degeneration focuses on the pathological alterations of the discs, accompanying imaging or laboratory biomarkers and symptoms. While the multifactorial etiology of the disease has become understood, little is known about the kinematics of the affected discs and adjacent spine structures.^79^

Under physiological conditions, spinal kinematics are influenced by the capacity of the discs to sustain external loading without marked alterations in their geometry. This capacity, known as mechanical stiffness, corresponds to a load–deformation curve, where bending of a functional unit (FU) is plotted as moment to displacement at each time point. This includes a neutral and an elastic zone. In the former deformation occurs against minimal internal resistance, while the in the latter linear resistance (stiffness) prevents deformation. The extent of the deformation affects the ROM of the segment, which is therefore an indicator of the overall stiffness of the disc.^65^ However, degenerated IVDs have lower height due to the dehydration of the NP and the disruption of the AF. Decreased disc height contributes to limited capacity to sustain mechanical loading without marked geometrical alterations. This renders the segment more prone to instability.

### Flexion/Extension

Segmental instability in the FE plane has been addressed by an observational clinical study^21^ (Fig. 6). Dai and colleagues, investigated the behavior of degenerated discs in lateral flexion and extension radiographs. Grade 1 and 2 degeneration of cervical IVDs were associated with 2 to 3 times higher extent cervical instability in comparison with Grade 3 and 4. Given that disc height is moderately decreased in moderate DD rather than in advanced DD, it appears that instability increases with slight to moderate disc height loss. Instability results to increased laxity of facet joints, which precipitates the formation of osteophytes limiting the segment's mobility. Therefore, it is reasonable to assume that in advanced degeneration the disruption of the disc and the development of osteophytes appear to limit the mobility of adjacent vertebrae. This behavior resembles the Kirkaldy-Willis degeneration pattern, where the initial stages of DD are characterized by increasing ROM due to instability, while further DD progression is accompanied by loss of flexibility.^43^

To the best knowledge of the authors, similar data about the behavior of degenerated stiff IVDs in LB and AR are not available.

#### Angular and Translational Range of Motion

Clinical studies based on MRI^33,56,57^ and radiographs^89^ have described the angular and translational ROM of the cervical spine in DD (Fig. 7). Miyazaki and colleagues have associated cervical angular and translational ROM with DD based on their own DD classification.^56^ The greatest translation and angular variation were observed in mild and moderate degeneration. In principle, mild and moderate degeneration was associated with more mobility, while severe degeneration was accompanied by a significant restriction, rendering the segment almost immobilized. In angular motion, the C3–C4 segment's mobility slightly deviated from this pattern being more mobile in severe rather than in early degeneration. It was significantly increased (approx. 29%) in moderate degeneration, and although severe degeneration restricted this motion by approximately 14%, the severely degenerated C3–C4 segment was still 15% more mobile when compared with its performance in early degeneration (Fig. 7a). Similarly, in translational motion, C5–C6 and C6–C7 were 15–20% more mobile in moderate and in severe degeneration compared to early stage degeneration. Progression from moderate to severe degeneration restricted translational motion by 36 and 7% respectively for C5–C6 and C6–C7 (Fig. 7b).

C4–C5 and C5–C6 segments had the greatest contribution to angular mobility, although this was decreased proportionally to the degree of degeneration.^56^ These findings are in line with the above mentioned physiological major contribution of C4–C6 to cervical ROM and with the higher frequency of DD occurring in these segments. Similar findings by Morishita and colleagues (2008),^57^ where C4–C6 were responsible for more than 45% of cervical ROM in mild and moderate DD. The contribution of the same segments in severe DD was reduced to 30 – 40% and was not compensated by adjacent segment. Instead, Occ-C1 level mobility is adjusted to compensate this.^33^ The greater overall motion of Occ-C1 in FE together with its configuration as the most cranial joint of the spine allows more freedom of motion available for compensation compared to C1–C2.^33^ Despite this, the progression of DD in the course of ageing results in a 32% reduction of the total cervical ROM and an approximately 1.5-fold increase of the C2–C7 lordotic angle between the 3rd and the 8th decade of life.^89^ Overall, while cervical DD progresses, cervical ROM is limited. Mild and moderate DD allow more motion, in expense of adjacent anatomical structures such as the facets and the bone endplates. Ultimately, the degeneration of these "compensatory" structures results in cervical ROM reduction and attachment of the cervical spine to a more lordotic configuration.

In a similar way to FE in the degenerative cervical spine, these alterations appear in line to Kirkaldy-Willis degeneration pattern, where DD progression marks a fluctuation from normal or even increased mobility to immobilization.^43^ Particular segments (C3–C4 for angular motion, C5–C7 for translational motion) remain mobile in severe DD. Although their mobility is restricted when compared to moderate degeneration, it is still higher than their physiological ROM. This can be partially be explained because these segments have a physiological significant contribution to the cervical ROM. The anatomical configuration of the majority of the remaining segments (Occ-C2 and C6–C7) restricts them from replacing the lost mobility. Therefore, it is possible that muscles and ligaments contribute to a degenerative equilibrium by supporting mobility in degenerated mobile segments. However, this is a mere assumption. The existing studies did not assess longitudinally whether mobility increased or decreased further in severe DD. Moreover, to the authors' best knowledge data regarding the degeneration of cervical paraspinal muscles and ligaments were not available to date.

Overall, the use of different DD grading systems and imaging methods renders a numerical comparison unreliable. Therefore, studies with comparable methodologies are needed to generate comparable evidence that can be used for a kinematic grading of cervical DD.

#### Center of Rotation

In the literature, the COR serves as an indicator of early—stage DD. Cadaveric studies suggest that the COR follows an unstable trajectory during the sagittal movement of degenerated segments.^67^ To the best of our knowledge, the cervical instant COR—ICR in DD has been investigated in one observational study based on FE and neutral radiographs of asymptomatic subjects and in one clinical study on patients undergoing anterior cervical discectomy and fusion and healthy control subjects. The former suggests that the mean ICR shifts anteriorly and higher in subjects with moderate and severe degeneration. Decreased disc space height, as a result of DD, seems to result in more antero-superior translation of the ICR.^51^ The clinical study has also shown small changes in the anterior location of the helical axis of motion in symptomatic motion segments. However, no such changes were detected in the adjacent cervical motion segments.^47^ Hence, it appears that DD mainly affects the rotational properties of the symptomatic segments.

### Spondylolisthesis

SL is defined as displacement of a vertebra in comparison to the physiological curve of the spine. The severity of SL is commonly assessed with the Meyerding grading system, which measures the percentage of translation of a vertebra compared to its subadjacent vertebra. Grades 1 to 4 stand for 0–25, 25–50, 50–75 and 75–100% respectively.^44^

**Figure 8:**
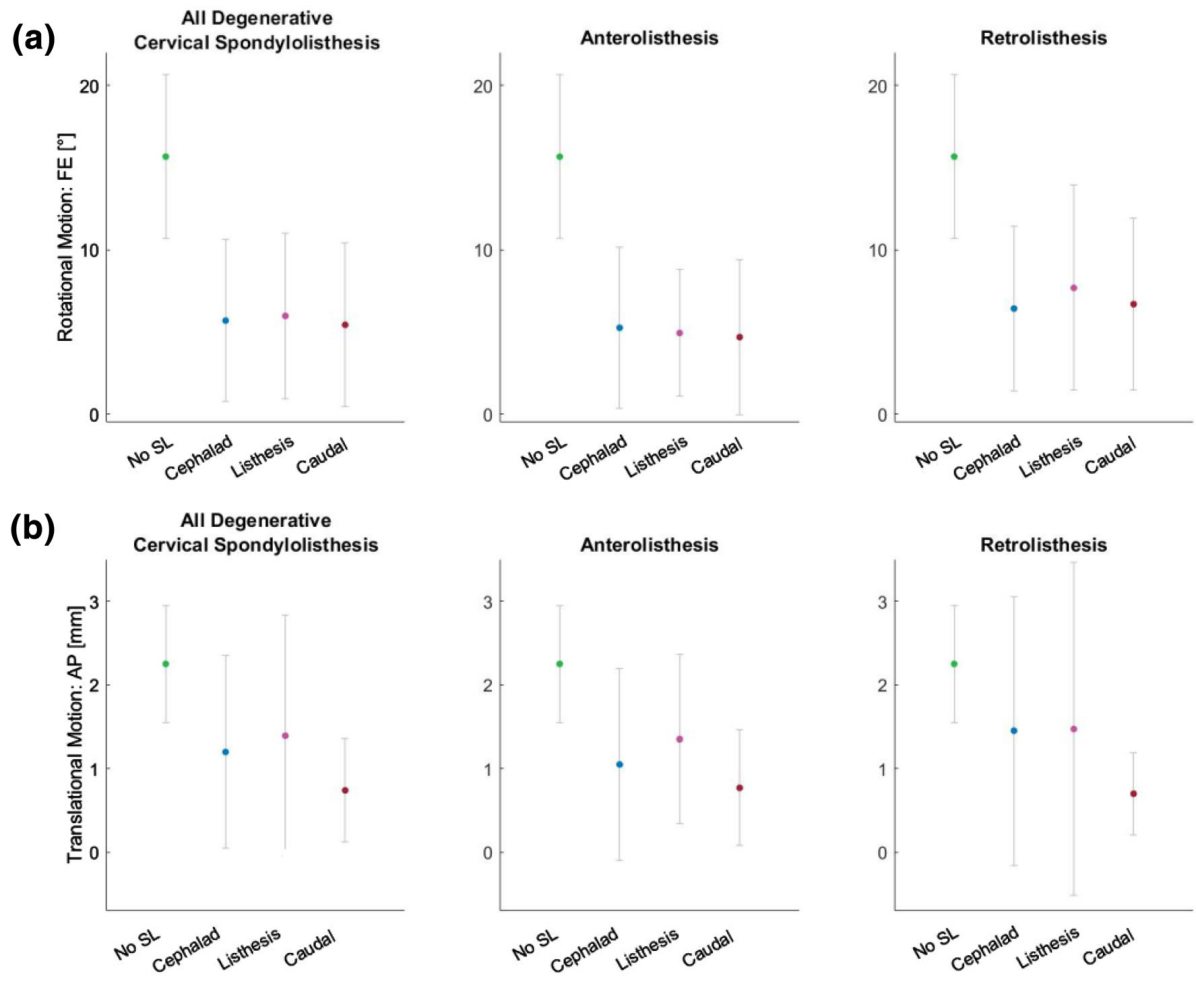
Rotational motion (a) and translational motion (b) for all types of degenerative cervical spondylolisthesis (DCS) or specified into anterolisthesis and retrolisthesis on the cephalad, listhesis or caudal level. No spondylolisthesis (No SL) corresponds to the average physiological ROM at C4–C6 segments as described in Fig. 2a. Those levels correspond to the levels with the highest frequency of disc degeneration. All illustrated data is from Paholpak *et al*.^62^

**Figure 9:**
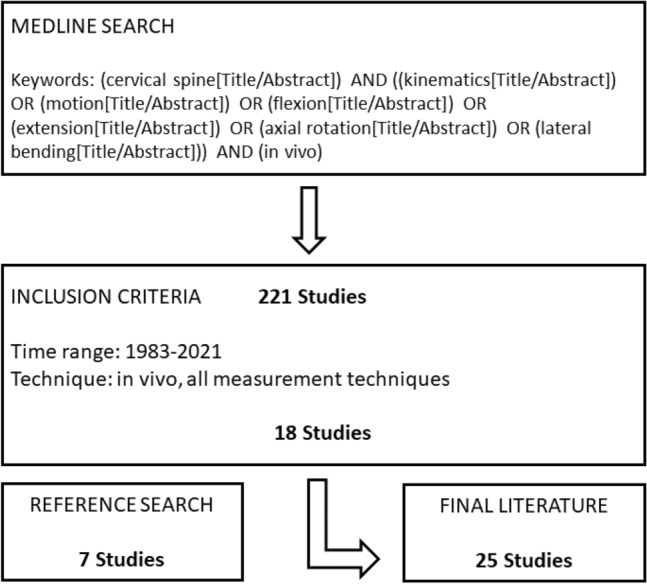
Search strategy for studies on segmental contribution in the cervical spine.

**Figure 10:**
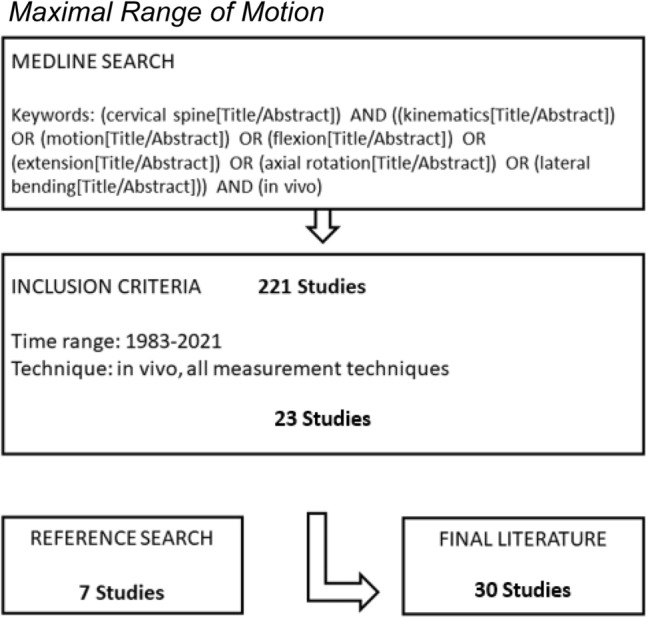
Literature search for studies on maximal ROM of the cervical spine.

**Figure 11:**
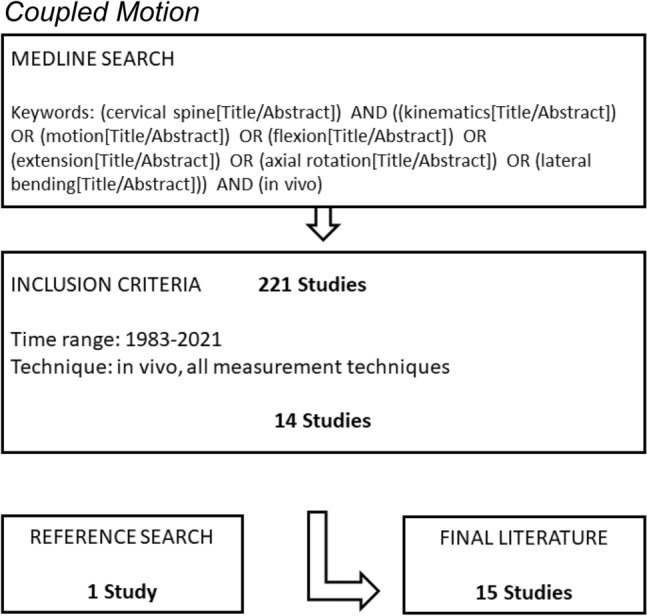
Literature search for studies on coupled angular motion and translation in the cervical spine.

**Figure 12:**
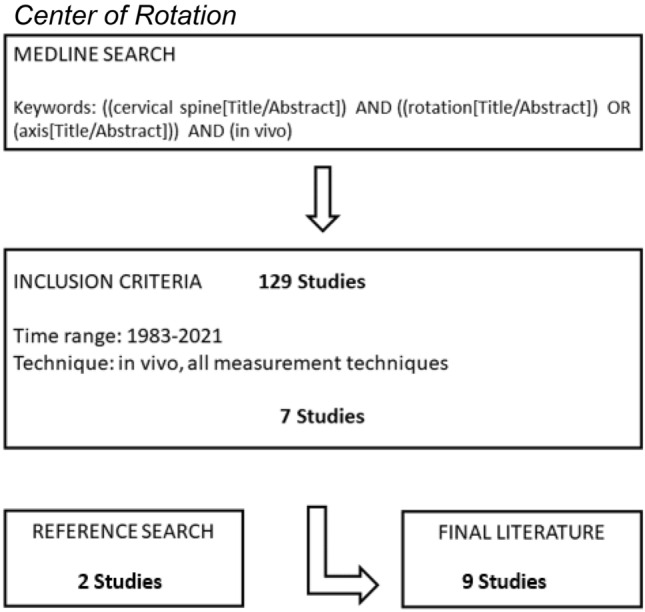
Literature search for studies on the center of rotation in the cervical spine.

**Figure 13:**
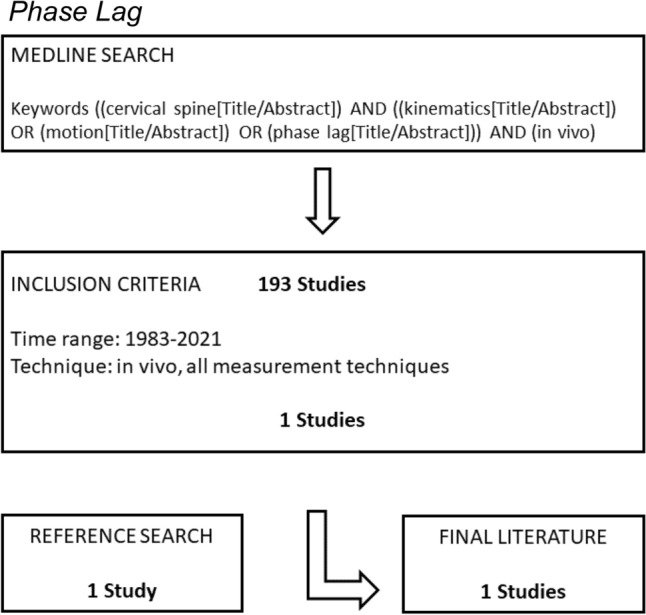
Literature search for studies on phase lag in the cervical spine.

**Figure 14:**
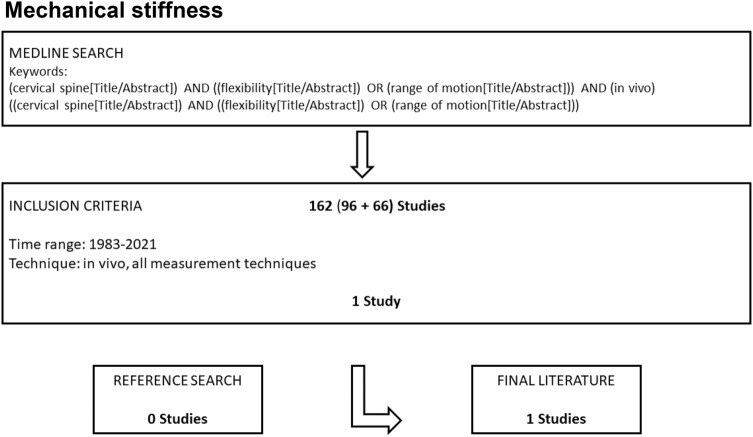
Literature search for studies on mechanical stiffness in cervical DD.

**Figure 15:**
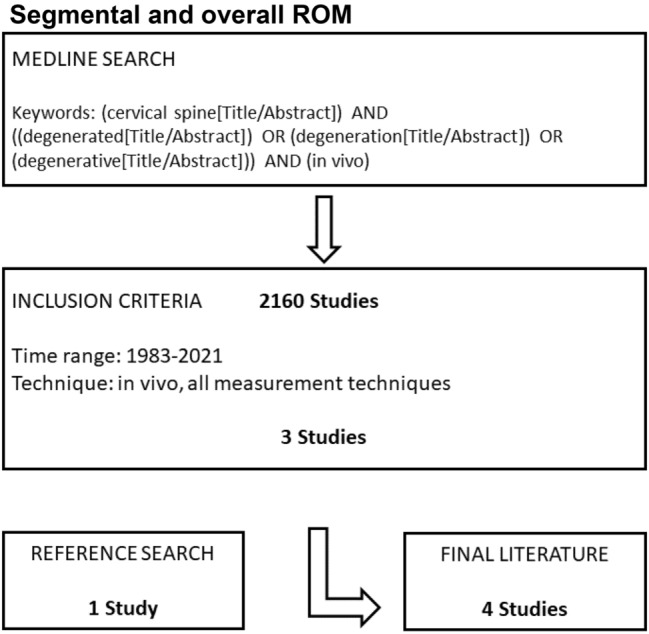
Literature search for studies on segmental and overall ROM in cervical DD.

**Figure 16:**
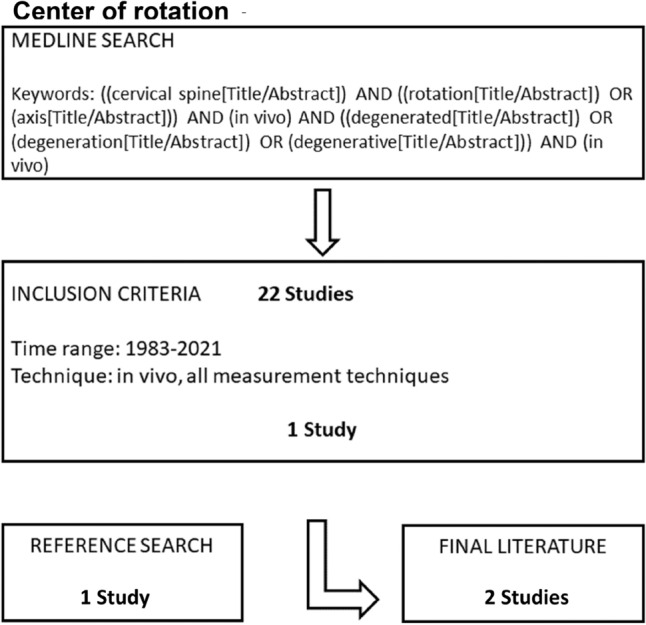
Literature search for studies on center of rotation in cervical DD.

**Figure 17:**
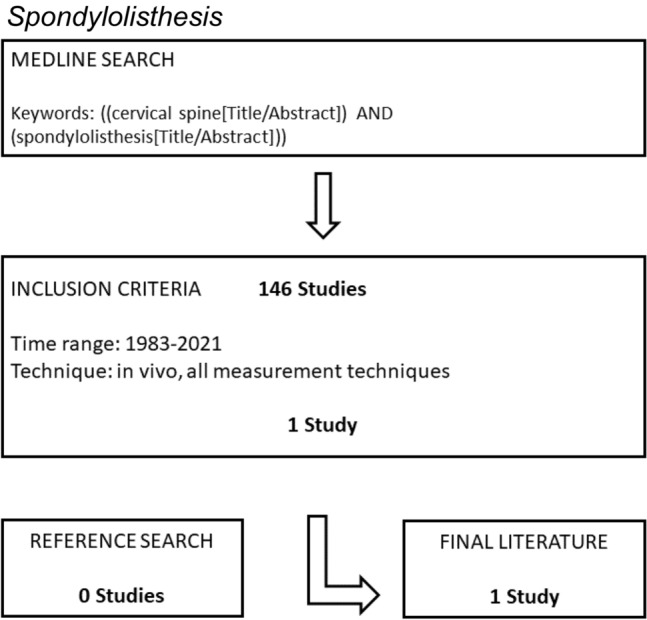
Literature search for studies on the effect of spondylolisthesis on the kinematics of the cervical spine.

Paholpak and colleagues (2017) studied SL of the cervical spine in an observational clinical study based on MRI data from approximately 1100 individuals^62^ (Fig. 8). The most commonly affected levels were C4-5 and C5-6, which physiologically have the greatest ROM in the cervical spine.^29,41,91,93^ Translation was greater at the cephalad level followed by the listhesis at the caudal level, particularly in individuals with overall degenerative cervical spondylolisthesis (DCS). The listhesis level had the highest rate of DD due to the constant exposure of the disc to abnormal loading. On the contrary, the cephalad level has the lowest rate of DD, which can be attributed to the maintenance of its translation. Overall, cervical SL leads to increased translation at the listhesis and the cephalad and caudal adjacent level and results in increased DD at the same levels.

When compared to the average physiological values, particularly at the most affected levels (C4–C6), segments with SL and their adjacent levels are less mobile.^83^ The displacement of the vertebrae outside the physiological spinal curve promotes muscular spasm and increased tension to the ligaments, leading to an altered motion pattern. The existing evidence suggests that this is a hypo-mobile pattern affecting not only the level of the slippage, but also its caudal and cephalad adjacent levels. On these grounds, dynamic imaging could help distinguish SL from moderate DD, where mobility is increased. Restricted mobility of the adjacent segments could also help differentiate SL from severe DD, in the sense that in the latter, adjacent structures and segments compensate for the lost mobility.^64^ Such a distinction would certainly be more challenging given the potential coexistence of SL and DD.

## Discussion

The cervical spine is the most mobile part of the vertebral column; therefore, its kinematics have particular significance in both healthy and degenerative conditions. In principle, the subaxial cervical spine bears most of FE and LB, while AR greatly depends on C1 – C2 (Fig. 1). FE ROM is mainly distributed between Occ-C1 and C3–C6, while LB ROM stems mostly from the subaxial cervical segments and AR ROM mainly depends on C1–C2 (Fig. 2). Motion is in most cases coupled with angular and translational mobility; rotation occurs more than translation from a quantitative point of view. In particular, AR exerts significant coupled FE angular motion at the Occ-C1 and the C7-Th1 levels, while FE and LB lead to moderate coupled angular motion in all planes across C2–C7 (Fig. 3). Simultaneously, FE results in significant coupled AP, SI and ML translation in the subaxial cervical spine, while LB and AR lead to minimal translation patterns, ranging between 1 and 2 mm across the same plane. The latter also confer notable SI translation at Occ-C1 level and significant AP and ML translation at C1–C2 level (Fig. 4). Motion migrates caudally from C1–C2 and at the same time COR translocates anteriorly and inferiorly within the respective vertebrae (Fig. 5).

In cervical spine degeneration, tissue damage and its pathomechanical sequelae modify the kinematic properties of the affected segments and their adjacent levels. Disc height reduction, listhesis and disproportionate loading of the facet joints and the bone endplates promote aberrant bone formation that decreases cervical mobility in patients with severe degeneration. A comparison between discs' mechanical stiffness (Fig. 5) and segmental ROM in DD (Fig. 6) is noteworthy. Mobility is increased in early and moderate DD and decreased in severe DD. Hence, it is reasonable to assume that early and moderate degenerative disc changes, reduce discs' stiffness enabling mobility. Conversely, in severe DD, dehydrated discs with minimal height are stiffer and restrict mobility.^57^ Certainly, mobility alone is not a measure of stiffness and these changes are also associated with osteophytes, bone endplates damage and the gradual adaptation of muscles and ligaments to the new motion pattern. In moderate DD, it is reasonable to assume that discs’ stiffness promotes abnormal motion due to rubbing and active compensation by paraspinal muscles. Subsequently, altered loading of bone endplates, facet joints, paraspinal muscles and ligaments inflicts damage on them as well. By the time, the discs are severely degenerated, and hence less stiff, these structures are unable to compensate movement.^76^ Therefore, mobility is significantly restricted. The fact that particular levels lose mobility in different stages of degeneration or even maintain considerable mobility under severe DD, indicates that degeneration is a heterogeneous process. A recent review has illustrated disc calcification as a phenotypic marker for the progression of DD.^90^ Differences in the cascade of trauma, hypoxia, inflammation, fibrosis and calcification may lead to distinct patterns of DD with variable kinematic implications. For this reason, severe degeneration can lead to segmental immobilization, restricted mobility or even increased mobility in different individuals. Attempting to associate this with particular kinematic changes and eventually devising kinematic biomarkers of DD could help personalize the management of cervical spinal degenerative disease.

However, to date and to the best knowledge of the authors, kinematics of the degenerative cervical spine have been reported by a limited number of studies focusing on DD and SL, additional studies encompassing other cervical degenerative conditions such as facet joint arthritis, ligament or paraspinal muscle degeneration might provide more comprehensive results about kinematic alterations in the course of degeneration. For the reported conditions, degeneration decreases mobility in all planes of motion. The degenerative process seems to initiate from the most mobile levels, particularly C4–C5 and C5–C6. Their ROM is equivocally affected in mild and moderate DD – this is also reflected by the anterosuperior transition of the ICR in the same DD stages. SL increases translation in the affected segment and the adjacent levels, in the expense of the disc whose degeneration paves the way to the aforementioned pathomechanical cascade.

Kinematic analysis of both the healthy and the degenerative spine would also benefit from common imaging methodologies. To date, the majority of the included studies were based on CT or MRI oftentimes combined with (video)fluoroscopy^29,50,80,88,93^ or biplanar radiographs.^6,7,9,11,88^ Fewer studies were based solely on radiography^27,35,71,75^ or fluoroscopy.^52,70,85^ The same variability of methods is evident in other studies reviewing spine kinematics.^28,74,83^ Previous studies on the matter have touched upon the differentiation of spinal kinematics in different configurations, such as walking^28^ or sit-to-stand movement,^69^ or in particular gait pathologies such as cervical myelopathy.^30^ To the best of the authors' knowledge, these studies are limited. Additional *in vivo* investigation with comparable methodologies are required, in order to map motion patterns in different healthy and pathological configurations in a systematic manner. The sitting and supine position are relevant in this regard.

To be able to draw conclusions about the reliability of the available measurements, one needs to acknowledge the inherent strengths and weaknesses of each imaging method. Earlier studies in *in vivo* spine kinematics have been based on radiographs. The accuracy of radiographs is low, because they allow only two – dimensional segmental motion analysis and they are not practical for measuring out – of plane rotations. Videofluoroscopy, magnetic resonance imaging (MRI) and computerized tomography (CT) have been introduced to measure three-dimensional (3D) spinal motions *in vivo*.^32,84^ Although their accuracy is comparable, the accuracy of CT reaches up to 0.1 mm in translation and 0.2° in rotation and appears superior comparing to other methods.^59^ Higher accuracy can be achieved with invasive stereo radiographic methods.^13^ Nevertheless, the risks of invasive methods and the radiation exposure due to CTs pose barriers for their investigational use in asymptomatic subjects. To the best of our knowledge, a head to head comparison between those methods and particularly MRI and videofluoroscopy in cervical spine kinematics has not been published. Indirect evidence come from clinical studies, where MRI is considered as the gold standard for the imaging of cervical curvature,^55^ while videofluoroscopy has high specificity for soft tissue damage.^26^ Therefore, in the frame of the present study, 3D – imaging (videofluoroscopy, MRI etc.), can be considered more reliable than radiographs. Variability between reference points for the detection of motion also poses significant challenges regarding the quantification of translational movement. Although the relevant studies used bone landmarks and captured the beginning and end of motion, specific aspects of the procedures vary. Older studies segmented motion components mannually^80^ or with semiautomatic manner,^35–37^ while more recent studies employed personalized volumetric models and either averaged all measurements or kept those trials where the beginning and the end of the movement were captured. The general agreement on recording the beginning and the end of the movement at anatomical osseous reference points enables comparison between the results, particularly in clusters of studies built on the same protocol.^2–11^ However, the study variations in recording and segmenting of motion planes should be taken into account together with the strengths and weaknesses of each imaging method. This reinstates the need for common methodology, if not broadly, at least with regard to particular planes of motion (angular, translational) or degenerative conditions.

Missing elements of knowledge include planes of motion not usually included in biomechanical studies and postoperative alterations of cervical kinematics. Neck circumduction constitutes a representative example of the former, where the neck performs a cross – like maneuver. Although circumduction is used for the clinical evaluation of neck pain,^94^ only few studies have investigated its kinematics. Currently, it is understood that circumduction combines elements of angular and translational motion in a distinct motion pattern. It is initiated and concluded with predominant FE (> 60% of the total motion), while LB and AR reach a peak of 40% of the total motion in the middle of right and left circumduction.^40^ In patients with neck pain, circumduction is characterized by aberrant movement (folds), which tend to decrease in the course of treatment.^23^ A number of studies providing evidence about different age and population groups and measuring aberrant movement across different causes of neck pain could shed more light to the matter. Similarly, different types of surgery can drastically reduce (fusion), attempt to maintain (arthroplasty, disc replacement) or even increase cervical motion (arthroplasty) at the operated and the adjacent cervical levels.^49,50^ Investigating the extent of kinematic alterations induced by iatrogenic interventions and compensatory activity of soft tissue and nerve control can help improve postoperative evaluation. Certainly, postoperative kinematics in different types of operations and patients' groups deem assessment in separate studies.

Additional concerns stem from averaging kinematic datasets across individuals of different age as well as data from multiple motion trials from the same individuals. This methodological design is quite common.^2–11^ It is reasonable to assume that this is a practical choice enabling researchers to collect and share as much information as possible. Including healthy subjects belonging to wide age ranges, from young adults to asymptomatic elderly, in the same investigation can help determine a range of physiological kinematic parameters that correspond to the general asymptomatic population regardless of age. This decreases the risk of considering the kinematic profile of an asymptomatic elderly or senior adult pathological, because it does not correspond to the range documented in younger adults, who tend to constitute the stereotype of a healthy population (i.e. a population with limited or no history of disease).

Nevertheless, averaging data from different age groups and/or including the same subjects in multiple trials is also a source of limitations. Currently, there is a consensus that the range of motion of the cervical spine decreases proportionally with increasing age.^66^ Therefore, it is possible that average estimations or lower limits of healthy/asymptomatic kinematics can be in fact misleading about the actual kinematic profile of younger adults. Potential clinical use of this knowledge can result in missing diagnoses among younger adults whose cervical kinematics are limited in comparison to their peers, but still fall within the physiological "one-size-for-all – age groups" spectrum. Conversely, averaging physiological angular or translation motion ranges to higher values because of the inclusion of young adults can lead to unnecessary diagnoses and investigations among senior adults or elderly. Sub – group analysis of the available and future datasets is necessary, in order to make the landscape of cervical kinematics more precise. In this frame, it is also important to pay attention to the median and distribution shape values of each age group, in order to understand the variation of kinematic properties across different body configurations or broader lifestyle and behavior patterns (eg white and blue collar workers, athletes and non – athletes). Spine diseases might also remain asymptomatic for years,^18^ before the onset of symptoms. Therefore, the definition of asymptomatic elderly and their inclusion into the healthy/asymptomatic group is also questionable. Similarly, the inclusion of the same subjects in multiple trials may confiscate the pragmatic nature of the collected results. The inclusion of elderly asymptomatic or "pre – symptomatic" subjects in multiple trials can result in a vicious circle of recycling shortcomings. Towards this end, diversifying study populations would be optimal. In case this is not possible due to the shortage of funding, facilities or volunteers, researchers should detect outlying parameters and adapt the statistical analysis accordingly.

Overall, in healthy/asymptomatic subjects, the extent and segmental distribution of translational motion in the AP, SI and ML planes are well described. The same applies to angular motion, both distinct and coupled. Evidence regarding the center of rotation and phase lag exists, but is limited. Additional studies might modify significantly the current perception of these kinematic parameters. More knowledge gaps exist when it comes to degenerative conditions. The majority of the available studies address translational and angular motion in DD. Only one study addresses SL. Evidence regarding the same in conditions such as facet joint osteoarthritis and tropism and paraspinal muscles and ligaments degeneration is scarce, if not absent. Center of rotation and phase lag are yet to be described in the frame of cervical spine degeneration. Therefore, more studies are needed for phase lag and center of rotation in both healthy and degenerative conditions, while broader investigation of the same is necessary for DD and SL. Particularly with regard to SL, the presence of solely one study can be misleading, if its results are not repeatable in future studies. Broader kinematic investigation is necessary for the non – studied diseases mentioned above. Ideally, future research should abide by common methodological approaches, provide comparable values and sub-group analyses and explore less – known, yet clinically relevant types of motion, such as circumduction.

## Supplementary Information

Below is the link to the electronic supplementary material.

Electronic supplementary material 1 (TIFF 91 kb)

Electronic supplementary material 2 (TIFF 90 kb)

Electronic supplementary material 3 (TIFF 87 kb)

Electronic supplementary material 4 (TIFF 80 kb)

Electronic supplementary material 5 (TIFF 82 kb)

Electronic supplementary material 6 (TIFF 92 kb)

Electronic supplementary material 7 (TIFF 93 kb)

Electronic supplementary material 8 (TIFF 84 kb)

Electronic supplementary material 9 (TIFF 93 kb)

## Appendix

### (Angular/Translational) Segmental Motion Contribution

See Fig. 9 and Table 1.

**Table 1:** Results of the literature search for studies for segmental contribution in the cervical spine.

Method	Subject number	Year	Author
CT + dual videofluoroscopy, dynamic, *active*, *in vivo*, LB + AR	10	2014	Lin^50^
CT, initial-final-position, *active, supine position*, *in vivo*, AR	20	2019	Kang^41^
MRI, incremental, *active, supine position*, *in vivo*, LB	12	2006	Ishii^36^
MRI + dual videofluoroscopy, dynamic, *active*, *in vivo*, FE + AR	10	2019	Yu^88^
CT + biplanar radiography, dynamic, *active*, *in vivo*, FE	18	2014	Anderst^6^
CT + dual videofluoroscopy, dynamic, *active*, *in vivo*, FE + LB + AR	10	2020	Zhou^93^
MRI, initial-final-position, *active, supine position*, *in vivo*, FE	15	2020	Muhlbauer^58^
CT, initial-final-position, *active, supine position*, *in vivo*, AR	16	2013	Zhao^91^
CT + biplanar radiography, dynamic, *active*, *in vivo*, FE + AR	10	2019	LeVasseur^48^
MRI, incremental, *active, supine position*, *in vivo*, AR	10	2004	Ishii^37^
CT/MRI + dual fluoroscopy, dynamic, *active*, *in vivo*, FE + LB + AR	18	2020	Wang^80^
CT + biplanar radiography, dynamic, *active*, *in vivo*, FE + LB + AR	29	2015	Anderst^7^
CT + biplanar radiography, dynamic, *active*, *in vivo*, FE	20	2013	Anderst^9^
CT + dual videofluoroscopy, dynamic, *active*, *in vivo*, AR	8	2021	Guo^29^
Radiography, initial-final-position, *active*, *in vivo*, FE	50	2008	Rousseau^71^
Fluoroscopy, dynamic, *active*, *in vivo*, FE	10	2007	Liu^52^
CT + biplanar radiography, dynamic, *active*, *in vivo*, FE + LB + AR	6	2016	Anderst^11^
CBCT, initial-final-position, *active*, *in vivo*, FE + LB + AR	20	2021	Tang^78^
Radiography, initial-final position, *in vivo*, *active*, FE	20	1999	Ordway^61^
Radiography, intital-final-position, *active*, *in vivo*, FE	41	2008	Simpson^75^
Radiography, initial-final position, *active*, *in vivo*, FE	137	2002	Frobin^27^
CT + biplanar radiography, dynamic, *active*, *in vivo*, FE	18	2013	Anderst^3^
Biplanar radiography, initial-final position, *active*, *in vivo*, AR	20	1993	Iai^35^
Fluoroscopy, dynamic, *active*, *in vivo*, FE	48	2010	Wu^85^
Fluoroscopy, dynamic, *active*, *in vivo*, FE	155	2004	Reitman^70^

#### Maximal Range of Motion

See Fig. 10 and Table 2.

**Table 2:** Results of the literature search for studies on maximal ROM of the cervical spine.

Method	Subject number	Year	Author
CT + dual videofluoroscopy, dynamic, *active*, *in vivo*, LB + AR	10	2014	Lin^50^
CT, initial-final-position, *active, supine position*, *in vivo*, AR	20	2019	Kang^41^
MRI, incremental, *active, supine position*, *in vivo*, LB	12	2006	Ishii^36^
MRI + dual videofluoroscopy, dynamic, *active*, *in vivo*, FE + AR	10	2019	Yu^88^
CT + biplanar radiography, dynamic, *active*, *in vivo*, FE	18	2014	Anderst^6^
CT + biplanar radiography, dynamic, *active*, *in vivo*, AR	20	2017	Anderst^10^
CT + dual videofluoroscopy, dynamic, *active*, *in vivo*, FE + LB + AR	10	2020	Zhou^93^
MRI, initial-final-position, *active, supine position*, *in vivo*, FE	15	2020	Muhlbauer^58^
CT, initial-final-position, *active, supine position*, *in vivo*, AR	16	2013	Zhao^91^
CT + biplanar radiography, dynamic, *active*, *in vivo*, FE + AR	10	2019	LeVasseur^48^
MRI, incremental, *active, supine position*, *in vivo*, AR	15	2004	Ishii^38^
MRI, incremental, *active, supine position*, *in vivo*, AR	10	2004	Ishii^37^
CT/MRI + dual fluoroscopy, dynamic, *active*, *in vivo*, FE + LB + AR	18	2020	Wang^80^
CT + biplanar radiography, dynamic, *active*, *in vivo*, FE + LB + AR	29	2015	Anderst^7^
CT + biplanar radiography, dynamic, *active*, *in vivo*, FE	20	2013	Anderst^9^
CT, initial-final-position, *active*, *in vivo*, FE + LB + AR	14	2021	Zhong^92^
CT + dual videofluoroscopy, dynamic, *active*, *in vivo*, AR	8	2021	Guo^29^
Radiography, initial-final-position, *active*, *in vivo*, FE	50	2008	Rousseau^71^
Fluoroscopy, dynamic, *active*, *in vivo*, FE	10	2007	Liu^52^
CT + biplanar radiography, dynamic, *active*, *in vivo*, FE + LB + AR	6	2016	Anderst^11^
CBCT, initial-final-position, *active*, *in vivo*, FE + LB + AR	20	2021	Tang^78^
Radiography, initial-final position, *active*, *in vivo*, FE + LB + AR	20	1978	Penning^68^
Radiography, initial-final position, *in vivo*, *active*, FE	20	1999	Ordway^61^
Videofluoroscopy, dynamic, *active*, *in vivo*, LB	46	1999	Lan^45^
Radiography, intital-final-position, *active*, *in vivo*, FE	41	2008	Simpson^75^
Radiography, initial-final position, *active*, *in vivo*, FE	137	2002	Frobin^27^
CT + biplanar radiography, dynamic, *active*, *in vivo*, FE	18	2013	Anderst^3^
Biplanar radiography, initial-final position, *active*, *in vivo*, AR	20	1993	Iai^35^
Fluoroscopy, dynamic, *active*, *in vivo*, FE	48	2010	Wu^85^
Fluoroscopy, dynamic, *active*, *in vivo*, FE	155	2004	Reitman^70^

#### Coupled Motion

See Fig. 11 and Table 3.

**Table 3:** Results of the literature search for studies on coupled angular motion and translation in the cervical spine.

Method	Subject number	Year	Author
CT + dual videofluoroscopy, dynamic, *active*, *in vivo*, LB + AR	10	2014	Lin^50^
CT, initial-final-position, *active, supine position*, *in vivo*, AR	20	2019	Kang^41^
MRI, incremental, *active, supine position*, *in vivo*, LB	12	2006	Ishii^36^
CT + dual videofluoroscopy, dynamic, *active*, *in vivo*, FE + LB + AR	10	2019	Zhou^93^
CT, initial-final-position, *active, supine position*, *in vivo*, AR	16	2013	Zhao^91^
MRI, incremental, *active, supine position*, *in vivo*, AR	15	2004	Ishii^38^
MRI, incremental, *active, supine position*, *in vivo*, AR	10	2004	Ishii^37^
CT/MRI + dual fluoroscopy, dynamic, *active*, *in vivo*, FE + LB + AR	18	2020	Wang^80^
CT + biplanar radiography, dynamic, *active*, *in vivo*, FE + LB + AR	29	2015	Anderst^7^
CT + biplanar radiography, dynamic, *active*, *in vivo*, FE	20	2013	Anderst^9^
CT, initial-final-position, *active*, *in vivo*, FE + LB + AR	14	2021	Zhong^92^
CT + dual videofluoroscopy, dynamic, *active*, *in vivo*, AR	8	2021	Guo^29^
CT + biplanar radiography, dynamic, *active*, *in vivo*, FE + LB + AR	6	2016	Anderst^11^
CBCT, initial-final-position, *active*, *in vivo*, FE + LB + AR	20	2021	Tang^78^
Biplanar radiography, initial-final position, *active*, *in vivo*, AR	20	1993	Iai^35^

#### Center of Rotation

See Fig. 12 and Table 4.

**Table 4:** Results of the literature search for studies on the center of rotation in the cervical spine.

Method	Subject number	Year	Author
CT + biplanar fluoroscopy, dynamic, *active*, *in vivo*, FE	3	2019	Kim^42^
MRI + dual videofluoroscopy, dynamic, *active*, *in vivo*, FE + AR	10	2019	Yu^88^
CT + biplanar radiography, dynamic, *active*, *in vivo*, FE	20	2013	Anderst^2^
MRI, initial-final-position, *active, supine position*, *in vivo*, FE	15	2020	Muhlbauer^58^
CT + biplanar radiography, dynamic, *active*, *in vivo*, FE	20	2021	Wawrose^81^
Radiography, initial-final-position, *active*, *in vivo*, FE	50	2008	Rousseau^71^
Radiography, initial-final-position, *active*, *in vivo*, FE	46	1991	Amevo^1^
Radiography, flexion—extension, *active*, *in vivo*, FE	59	1983	Dvorak^22^
Radiography, initial-final-position, *active*, *in vivo*, FE	39	2012	Barrey^15^

#### Phase Lag

See Fig. 13 and Table 5.

**Table 5:** Results of the literature search for studies on phase lag in the cervical spine.

Method	Subject number	Year	Author
Cineradiography, dynamic, *active*, *in vivo*, FE	10	1999	Hino^34^

### Disc Degeneration

#### Mechanical stiffness

See Fig. 14 and Table 6.

**Table 6:** Results of the literature search for studies on mechanical stiffness in cervical DD.

Method	Specimen number	Year	Author
MRI + biplanar radiography, initial-final-position, *active*, *in vivo*, FE	260	1998	Dai^21^

#### Segmental and overall ROM

See Fig. 15 and Table 7.

**Table 7:** Results of the literature search for studies on segmental and overall ROM in cervical DD.

Method	Subject number	Year	Author
kMRI, initial-final-position, *active*, *in vivo*, FE	164	2008	Miyazaki^56^
kMRI, initial-final-position, *active*, *in vivo*, FE	289	2008	Morishita^57^
Single plane radiography, initial-final-position, *active*, *in vivo*, FE	1230	2012	Yukawa^89^
kMRI, initial-final position, *active*, FE	446	2016	Hayashi^33^

#### Center of rotation

See Fig. 16 and Table 8.

**Table 8:** Results of the literature search for studies on center of rotation in cervical DD.

Method	Subject number	Year	Author
Single plane radiography, initial-final-position, *active*, *in vivo*, FE + LB	680	2014	Liu^51^
Synchronized Biplanar radiography, *active*, *in vivo*, FE	100	2022	LeVasseur

#### Spondylolisthesis

See Fig. 17 and Table 9.

**Table 9:** Results of the literature search for studies on the effect of spondylolisthesis on the kinematics of the cervical spine.

Method	Subject number	Year	Author
kMRI, *active*, *in vivo*, seated FE + weight bearing neutral	1128	2017	Paholpak^62^

## Abbreviations

AOR: Axis of rotation
AR: Axial rotation
COR: Center of rotation
DD: Disc degeneration
DCS: Degenerative cervical spondylolisthesis
FE: Flexion/extension
FJOA: Facet joint osteoarthritis
FJT: Facet joint tropism
FU: Functional unit
IL: Interspinous ligament
LB: Lateral bending
LD: Ligament degeneration
LF: Ligamentum flavum
LFH: Ligamentum flavum hypertrophy
NZ: Neutral zone
OA: Osteoarthritis
PMD: Paraspinal muscle degeneration
ROM: Range of motion
SL: Spondylolisthesis
