# Supplementary figures and images for: Kinematics of the Cervical Spine Under Healthy and Degenerative Conditions: A Systematic Review
Source: Ann Biomed Eng. 2022 Dec 10;50(12):1705–33. doi: 10.1007/s10439-022-03088-8 (PMC9794546; doi:10.1007/s10439-022-03088-8)

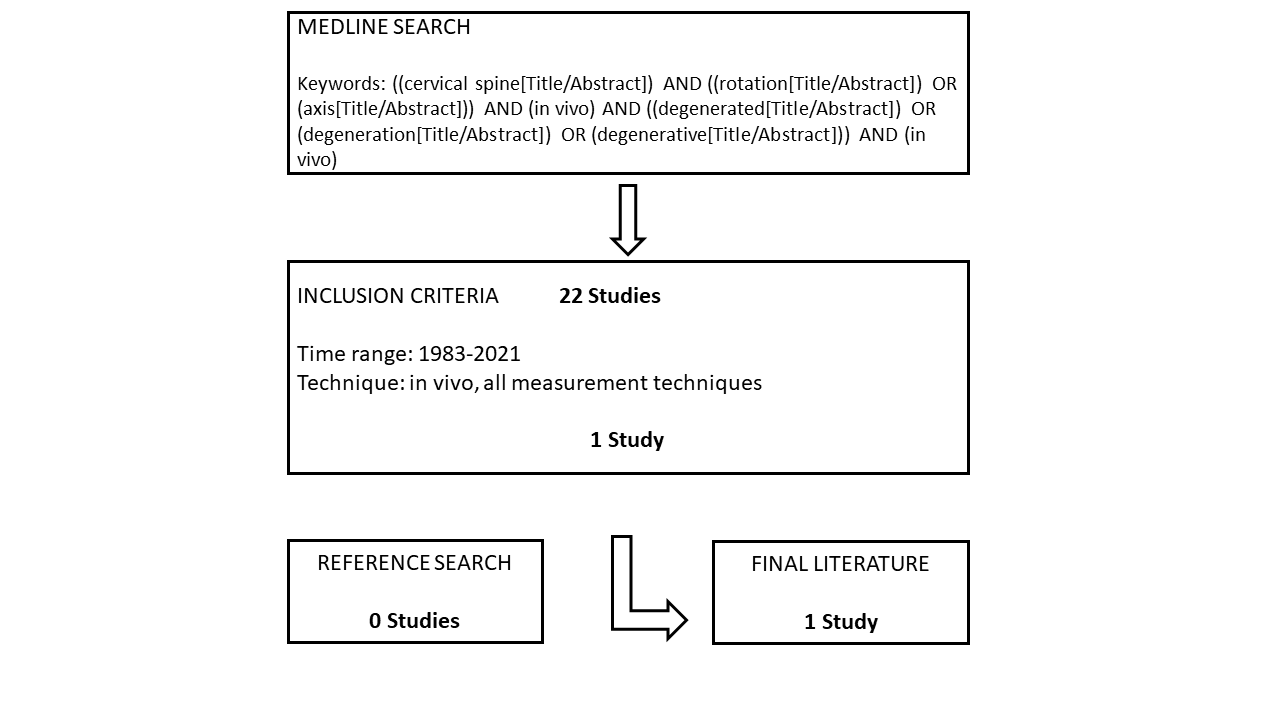

Supplement: Supplementary file 1 — Electronic supplementary material 1 (TIFF 91 kb) [file 10439_2022_3088_MOESM1_ESM.tiff]

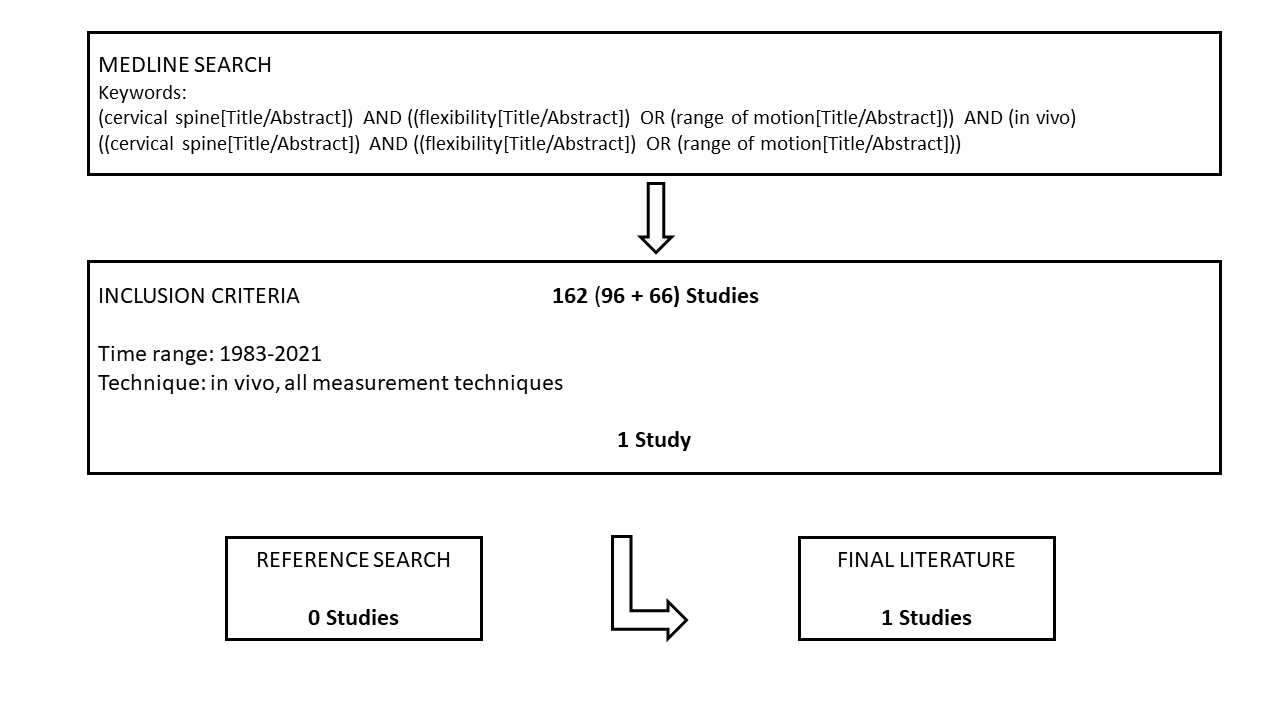

Supplement: Supplementary file 2 — Electronic supplementary material 2 (TIFF 90 kb) [file 10439_2022_3088_MOESM2_ESM.tiff]

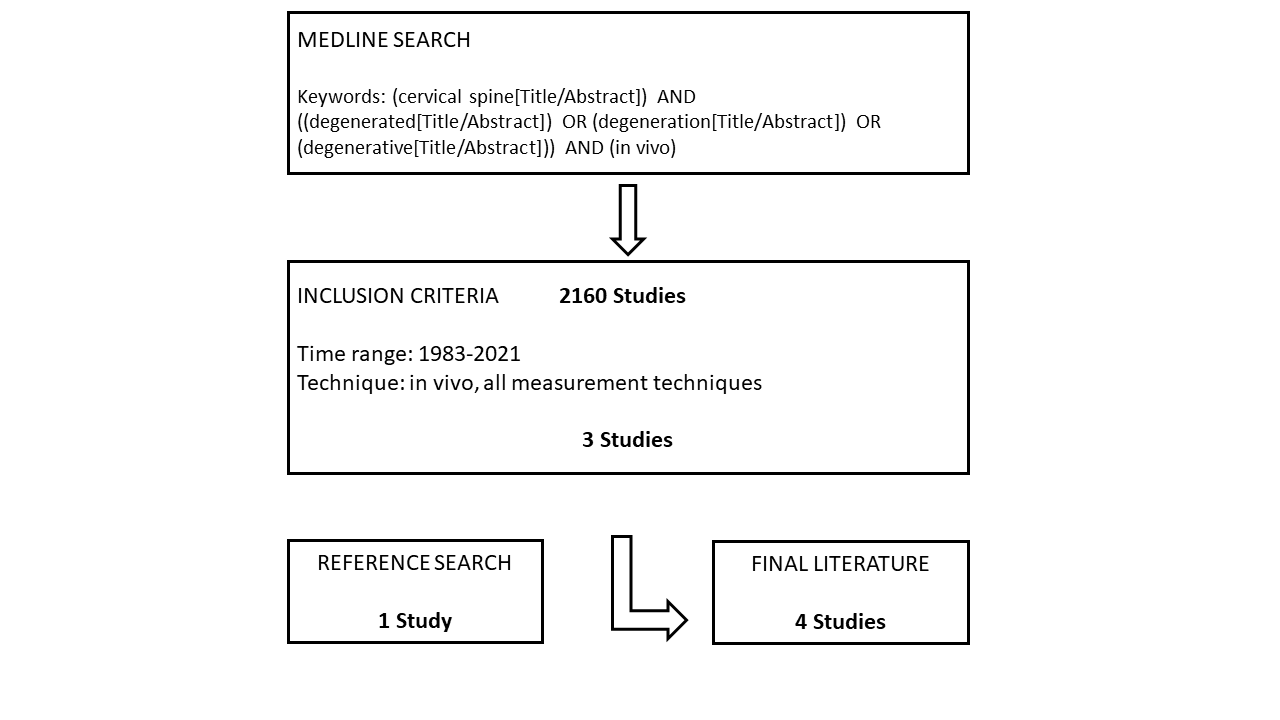

Supplement: Supplementary file 3 — Electronic supplementary material 3 (TIFF 87 kb) [file 10439_2022_3088_MOESM3_ESM.tiff]

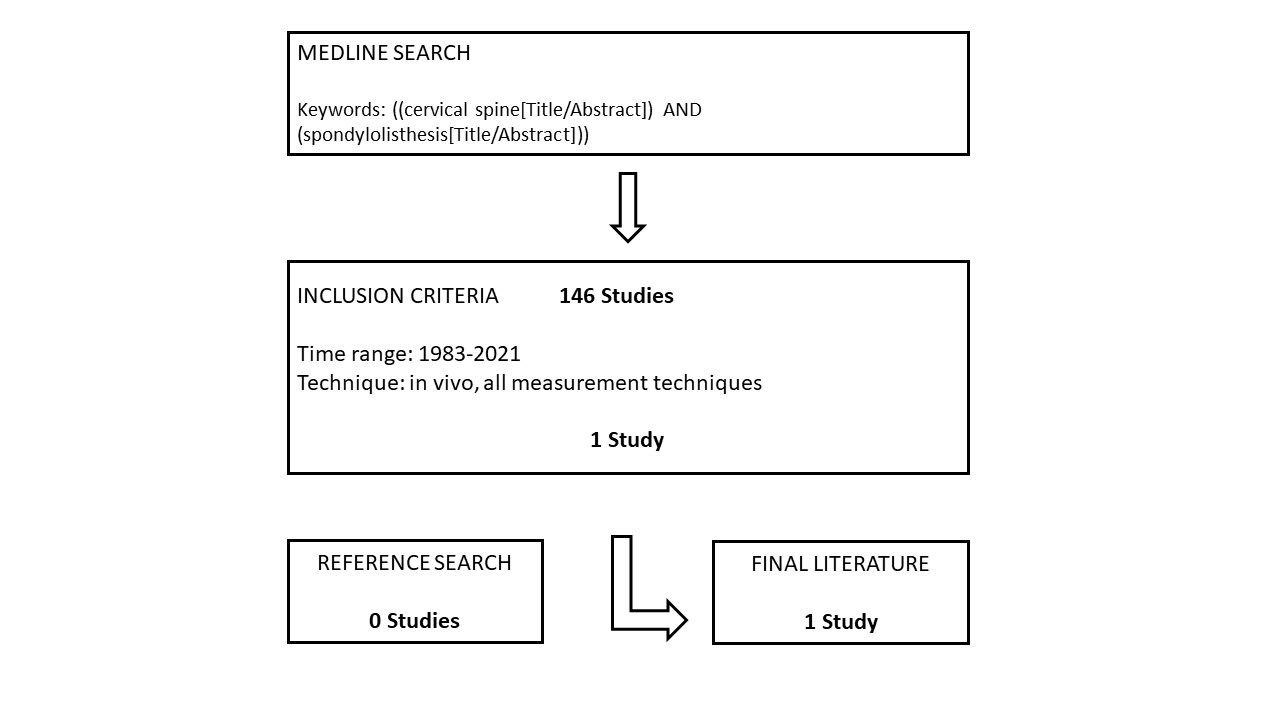

Supplement: Supplementary file 4 — Electronic supplementary material 4 (TIFF 80 kb) [file 10439_2022_3088_MOESM4_ESM.tiff]

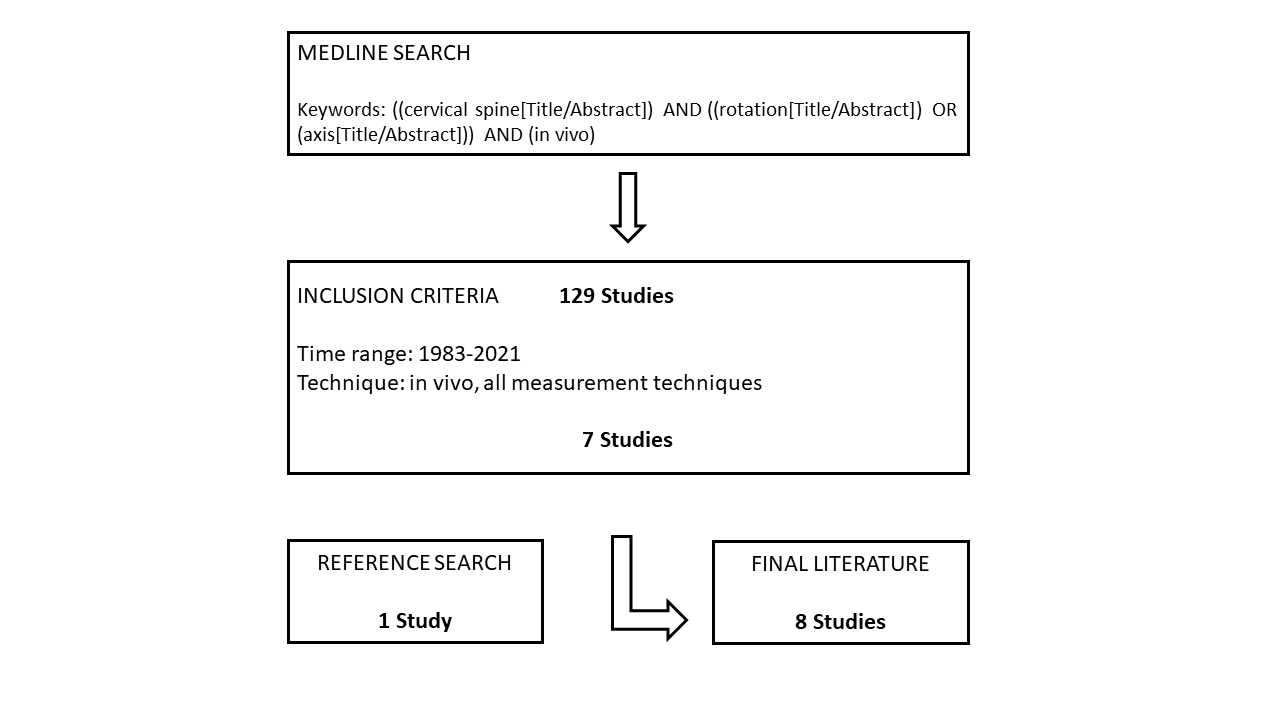

Supplement: Supplementary file 5 — Electronic supplementary material 5 (TIFF 82 kb) [file 10439_2022_3088_MOESM5_ESM.tiff]

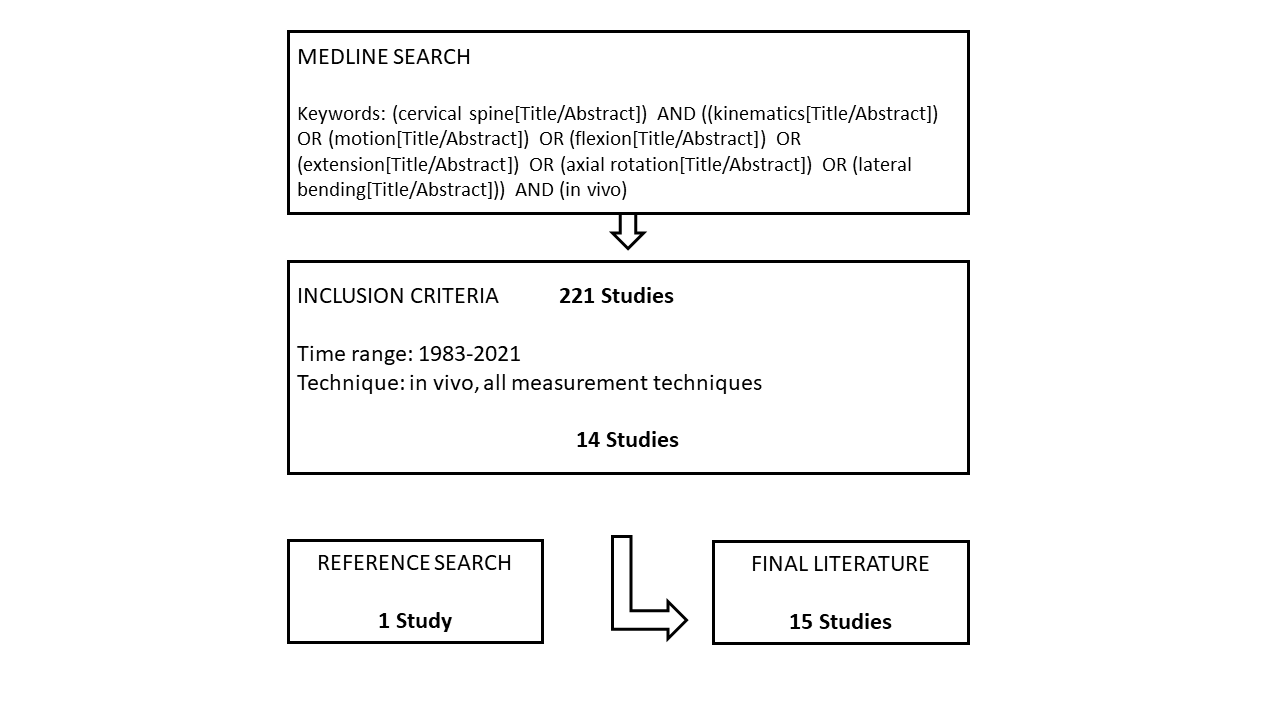

Supplement: Supplementary file 6 — Electronic supplementary material 6 (TIFF 92 kb) [file 10439_2022_3088_MOESM6_ESM.tiff]

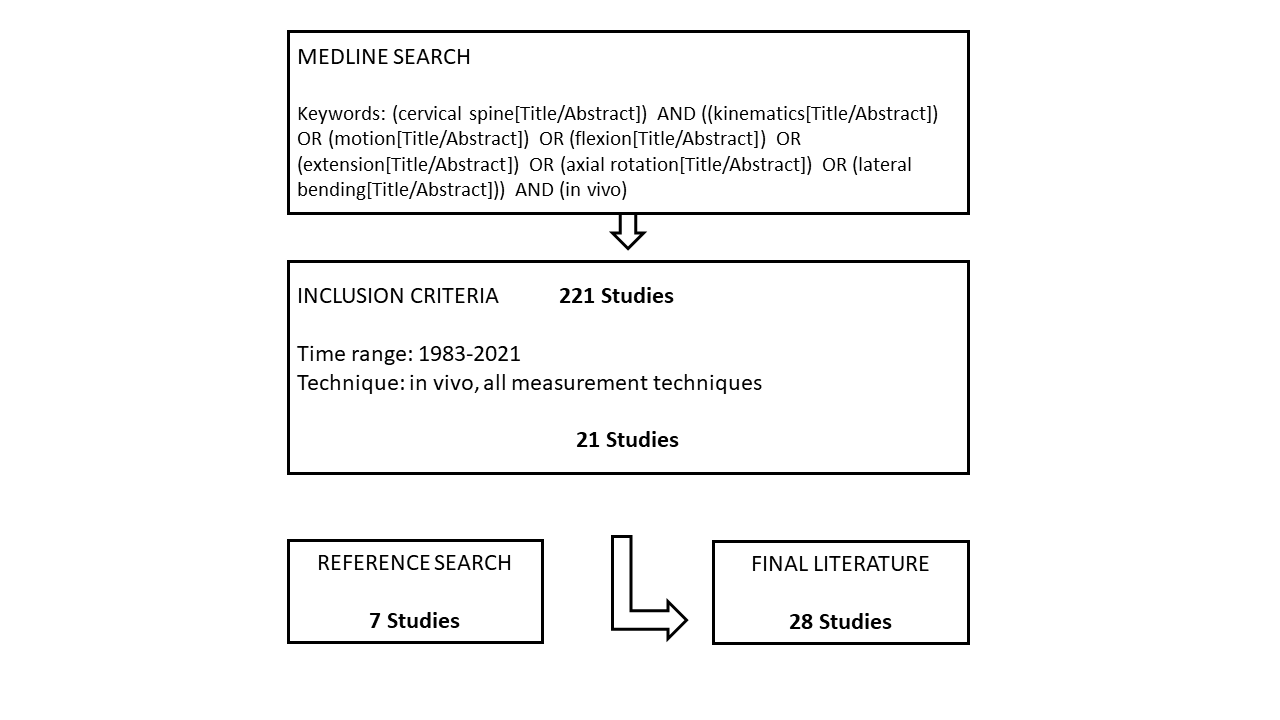

Supplement: Supplementary file 7 — Electronic supplementary material 7 (TIFF 93 kb) [file 10439_2022_3088_MOESM7_ESM.tiff]

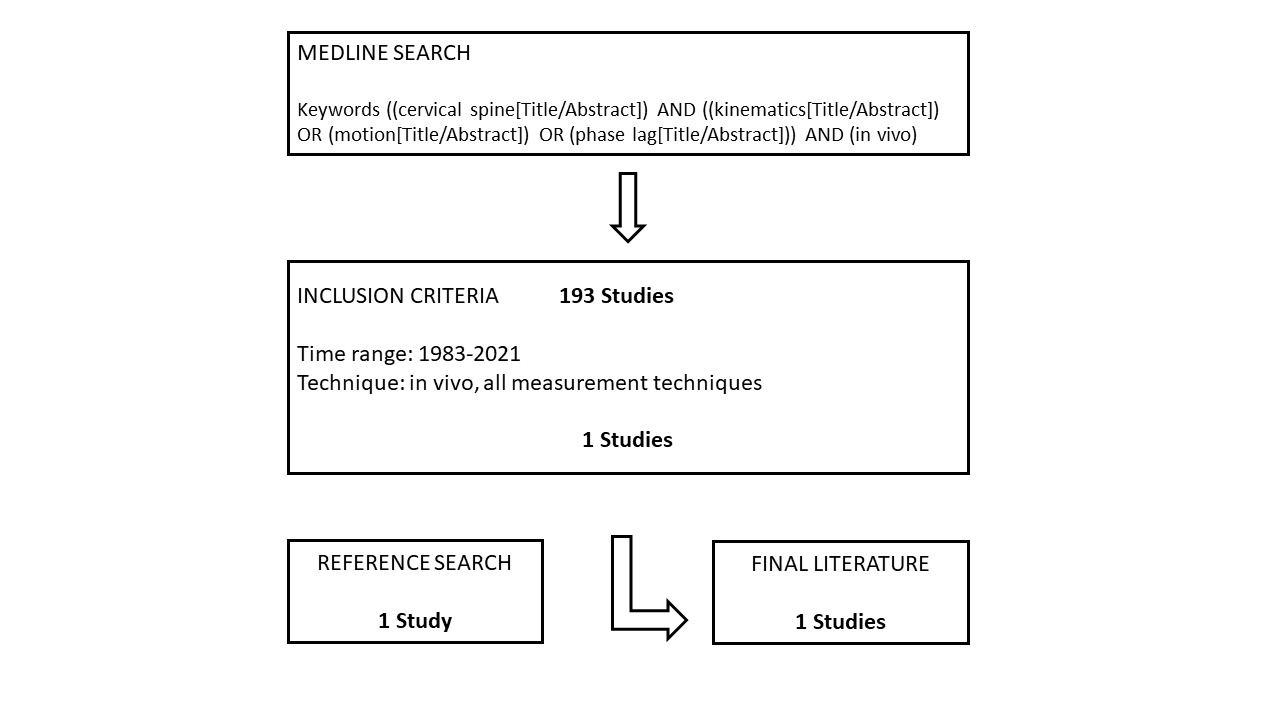

Supplement: Supplementary file 8 — Electronic supplementary material 8 (TIFF 84 kb) [file 10439_2022_3088_MOESM8_ESM.tiff]

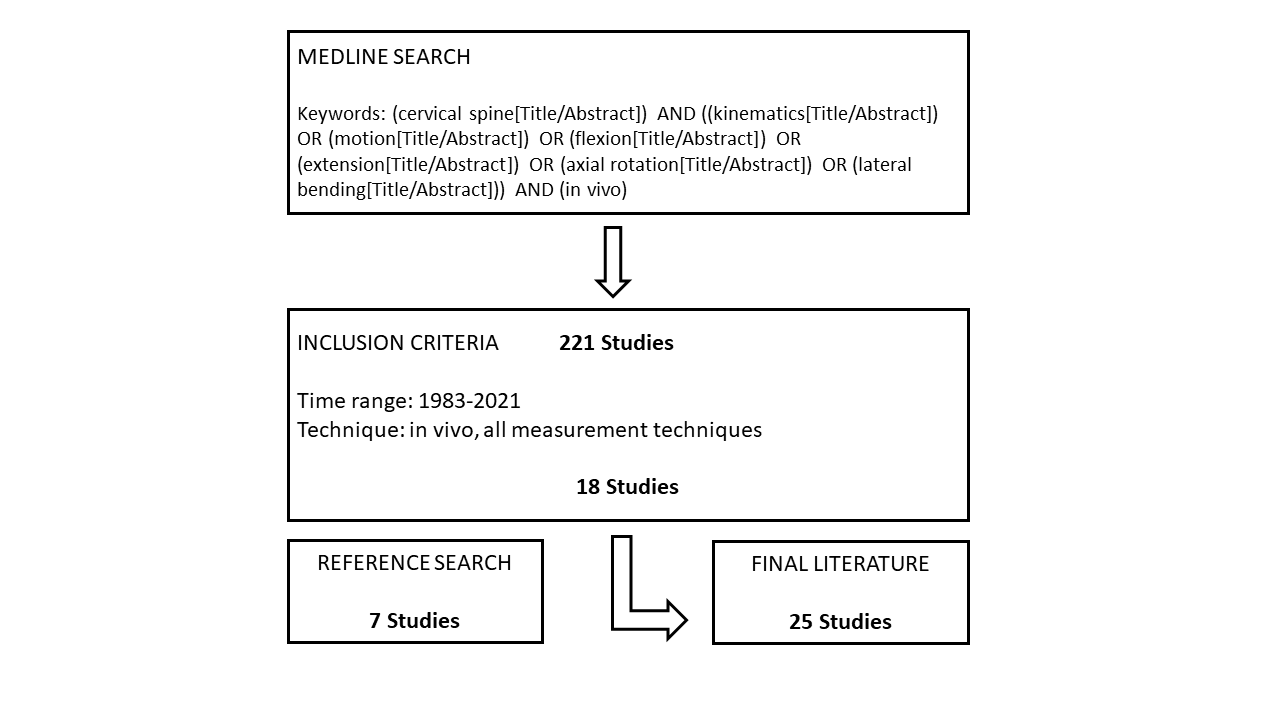

Supplement: Supplementary file 9 — Electronic supplementary material 9 (TIFF 93 kb) [file 10439_2022_3088_MOESM9_ESM.tiff]
